# Exploring Mechanisms of Hydration and Carbonation of MgO and Mg(OH)_2_ in Reactive Magnesium Oxide-Based Cements

**DOI:** 10.1021/acs.jpcc.1c10590

**Published:** 2022-03-30

**Authors:** Mina Ghane Gardeh, Andrey A. Kistanov, Hoang Nguyen, Hegoi Manzano, Wei Cao, Paivo Kinnunen

**Affiliations:** † Fibre and Particle Engineering Research Unit, University of Oulu, Pentti Kaiteran katu 1, 90014 Oulu, Finland; ‡ Nano and Molecular Systems Research Unit, University of Oulu, Pentti Kaiteran katu 1, 90014 Oulu, Finland; § Departament of Condensed Matter Physics, University of the Basque Country (UPV/EHU), Barrio Sarriena, s/n, 48940 Leioa, Spain

## Abstract

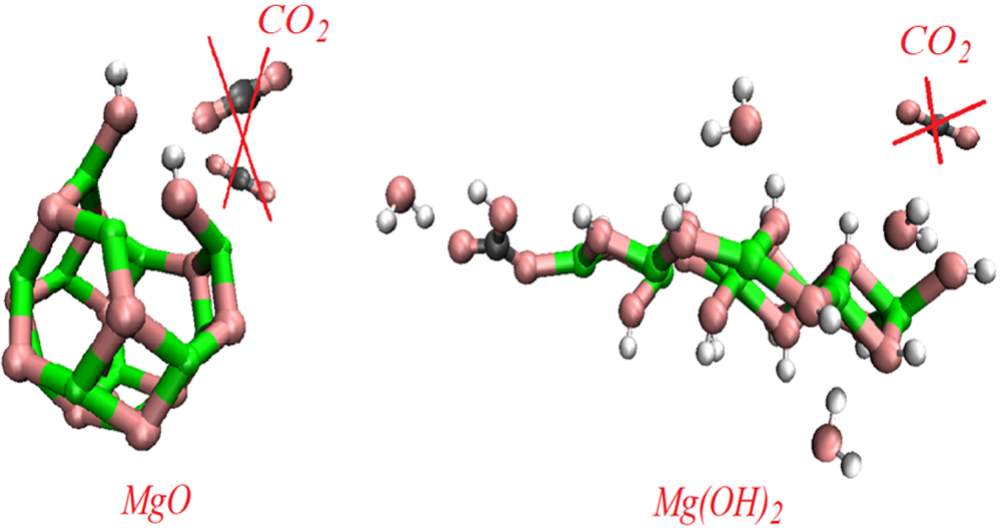

Reactive magnesium oxide (MgO)-based cement (RMC) can play a key role in carbon capture processes. However, knowledge on the driving forces that control the degree of carbonation and hydration and rate of reactions in this system remains limited. In this work, density functional theory-based simulations are used to investigate the physical nature of the reactions taking place during the fabrication of RMCs under ambient conditions. Parametric indicators such as adsorption energies, charge transfer, electron localization function, adsorption/dissociation energy barriers, and the mechanisms of interaction of H_2_O and CO_2_ molecules with MgO and brucite (Mg(OH)_2_) clusters are considered. The following hydration and carbonation interactions relevant to RMCs are evaluated: (i) carbonation of MgO, (ii) hydration of MgO, carbonation of hydrated MgO, (iii) carbonation of Mg(OH)_2_, (iv) hydration of Mg(OH)_2_, and (v) hydration of carbonated Mg(OH)_2_. A comparison of the energy barriers and reaction pathways of these mechanisms shows that the carbonation of MgO is hindered by the presence of H_2_O molecules, while the carbonation of Mg(OH)_2_ is hindered by the formation of initial carbonate and hydrate layers as well as presence of excessed H_2_O molecules. To compare these finding to bulk mineral surfaces, the interactions of the CO_2_ and H_2_O molecules with the MgO(001) and Mg(OH)_2_ (001) surfaces are studied. Therefore, this work presents deep insights into the physical nature of the reactions and the mechanisms involved in hydrated magnesium carbonates production that can be beneficial for its development.

## Introduction

Increasing carbon dioxide (CO_2_) emissions are currently one of the most serious environmental challenges.^1^ Cement manufacturing, and specifically the manufacture of ordinary Portland cement (OPC), is the source of ∼5%–7% of global greenhouse gas emissions.^2^ Limestone (CaCO_3_), the conventional feedstock for OPC manufacturing, is excavated, crushed, and sintered with other materials in a cement kiln at temperatures reaching ∼1450 °C to produce clinker. During the calcination of CaCO_3_, CO_2_ is directly emitted (i.e., CaCO_3_ → CaO + CO_2_), causing ∼50%–60% of the total emissions from OPC production.^3^ From the standpoint of sustainable development, the cement industry is seeking alternatives to reduce CO_2_ emissions while maintaining the same performance.^4^

Among the proposed alternative binders, Mg-based cements have attracted attention for their promise as partial replacements for OPC.^5^ When magnesium oxide (MgO) is derived from Mg silicates (e.g., olivine and serpentine), less environmental and economic impact is generated.^6^ The net CO_2_ emissions from the carbonation of these binders may be ∼73% lower than OPC^7^ and, therefore, may potentially lead to the formation of carbon-negative cements. Moreover, the lower production temperature of reactive MgO-based cement (RMC) compared to that of OPC (i.e., 700–1000 °C vs 1450 °C), and its potential to gain strength through its reaction with CO_2_, have attracted special attention.^7^

Considering the need for the rapid development of carbon capture and utilization technology,^8^ the main advantage of RMCs produced from Mg–Si minerals in concrete formulations is their ability to absorb and permanently store CO_2_ in the form of stable carbonates during the carbonation process when MgO is sourced from low-CO_2_ feedstocks.^9^ In such processes, MgO reacts with water (H_2_O) to form brucite (Mg(OH)_2_), which generally has a weak and porous structure.^5,10^ However, hydrated MgO has a strong ability to absorb CO_2_ and produce carbonated products at a strength useful for construction purposes.^11^ In other words, the dissolution of MgO through hydration results in the formation of Mg(OH)_2_, which is then carbonated according to the following reaction and produces a range of hydrated magnesium carbonates (HMCs): Mg(OH)_2_ + CO_2_ + 2H_2_O → MgCO_3_·3H_2_O. Nesquehonite (MgCO_3_·3H_2_O) is the most commonly obtained HMC, yet other phases such as hydromagnesite (4MgCO_3_·Mg(OH)_2_·4H_2_O), dypingite (4MgCO_3_·Mg(OH)_2_·5H_2_O), and artinite (MgCO_3_·Mg(OH)_2_·3H_2_O) can also be present.^12,13^

Recent experimental studies have examined the formation of HMCs through the hydration and carbonation of RMC. In particular, improvement of the hydration and mechanical performance of carbonated MgO-based systems has been observed with the introduction of various hydration agents at different concentrations.^14^ In this way, the simultaneous use of magnesium acetate at 0.05 M and carbonate seeds (up to 1% of cement content) improved mechanical performance of carbonated RMC concrete mixes.^15^ However, investigation of the physical nature of mechanisms involved in the reactions of HMC production is still immature. One of the reasons for this is the limitation of available experimental methods for the determination of such processes occurring at the nanoscale in bulk materials.

Theoretical approaches with predictive capabilities, such as those based on the density functional theory (DFT), show a high capability for determining the most stable atomic structures and exploring the physical and chemical properties of these finite systems.^16−19^ Computational approaches have been successfully utilized to investigate in depth the mechanisms related to the formation of HMCs. For instance, the structure, formation energy, and electronic properties of four commonly exposed surfaces of nesquehonite crystal have been studied by using DFT-based calculations.^16^ In another computational work the activity and selectivity of MgO surfaces for CO_2_ conversion have been studied.^20^ In particular, the adsorption and dissociation of CO_2_, as well as its subsequent hydrogenation to HOCO and HCOO, on various MgO surfaces, have been investigated. It has been shown that the direct dissociation of CO_2_ on MgO is thermodynamically unfavorable because of high reaction energy, while hydrogenation of CO_2_ to HCOO by hydride H is more feasible on MgO. DFT simulations have also been utilized to compare the adsorption and activation reaction mechanisms of CO_2_ and H_2_ molecules on hydrogen-assisted MgO(110), pure Ni(111), and Ni/MgO interfaces.^21^ Computational methods have also been applied for a deeper exploration of the effects of various promoters and dopants upon CO_2_ adsorption on the MgO–CaO(100) surface.^22^ Theoretically supported experimental infrared-based studies have been performed to identify the structure of the CO_2_ species adsorbed on the various MgO surface.^23^ It has been shown that the active site toward CO_2_, which is a Lewis acid, differs from that for the deprotonating adsorption of Brønsted acids. Another experimentally supported computational study provided a comprehensive study of the CO_2_ adsorption on the MgO and Mg(OH)_2_ surfaces.^24^ It has been found that chemisorption of CO_2_ on the MgO surface is facilitated by the presence of H_2_O.

Because the reaction degrees of MgO and Mg(OH)_2_ are relatively low (ca. 50%), they reduce the effectiveness of CO_2_ utilization to form a cementitious binder.^25^ Furthermore, because the transformation of HMCs shows mixed diffusion and reaction-limited control, and it proceeds through the production of metastable intermediates, the specifics of nesquehonite conversion to other HMCs remain unclear. The conversion of these metastable intermediates also raises concerns about the durability of cement.^26^ Therefore, insights into the potential reactions in the MgO/H_2_O/CO_2_ system, and an understanding of the nature of kinetic hindrance in MgO and Mg(OH)_2_ carbonation and hydration at the atomic level, are of immediate interest.

In this work, the physical nature of the mechanisms for HMC production on MgO and Mg(OH)_2_ nanoclusters is considered by using DFT calculations. Clusters are collections of atoms that act as a link between gases and bulk phase materials (liquids and solids). They are considerably large to be considered as molecules while considerably small to be classified as liquids or solids, and almost all of the atoms in a cluster are on or near its surface, making them a good choice for considering surface reactions.^27^ In addition, robust reactions at oxide surfaces, such as the exchange rates of H_2_O molecules on the surface, can be reliably predicted by using molecular simulation methods.^28^

Here, the interaction of these nanoclusters of potentially promising RMC raw materials with ambient molecules (H_2_O and CO_2_) is considered. The mechanism of the following reactions is investigated: carbonation of MgO, hydration of MgO, carbonation of hydrated MgO, carbonation of Mg(OH)_2_, hydration of Mg(OH)_2_, and hydration of carbonated Mg(OH)_2_. Notably, even though through-solution dissolution–precipitation reactions are often the dominating reactions in HMC synthesis, surface carbonation can become important to the overall carbonation kinetics by hindering further reactions, including dissolution. Understanding the mechanisms of these reactions is accomplished by calculating adsorption energy, charge transfer, electron localization function, and adsorption/dissociation energy barriers of H_2_O and CO_2_ upon reactions with the MgO and Mg(OH)_2_ clusters. To gain further insights into the difference between MgO and Mg(OH)_2_ clusters and bulks, the interactions between the surfaces of bulk MgO and Mg(OH)_2_ with H_2_O and CO_2_ molecules are also investigated. The results also shed light on the underlying reason for the hindrance of carbonation of MgO and Mg(OH)_2_ that has been previously observed experimentally. Therefore, the results of this work reveal the mechanisms that take place during HMC production that can further facilitate the development of their production.

## Methods

The calculations were performed based on DFT using the Vienna ab initio simulation package^29^ where the electron–ion interactions were simulated via the projector augmented wave method.^30^ The generalized gradient approximation with the Perdew–Burke–Ernzerhof exchange-correlation function was employed.^31^ The most energetically favorable MgO cluster has a cage-like configuration with *T*_*h*_ symmetry that included six Mg_2_O_2_ rings and eight Mg_3_O_3_ to form a shortened octahedron with equivalent Mg and O vertices.^32^ The system considered consisted of a MgO cluster placed in a cubic supercell with dimensions of 20 × 20 × 20 Å. A 3 × 3 × 3 *k*-point sampling was employed for structure optimization calculations, while a 1 × 1 × 1 *k*-point was used for electronic structure calculations. Mg(OH)_2_ cluster consisting of nine units of Mg(OH)_2_^33^ was placed in a cubic cell with dimensions of 30 × 30 × 30 Å. A 1 × 1 × 1 Å *k*-point sampling was applied for all optimization and electronic structure calculations. The considered MgO and the Mg(OH)_2_ slabs with the (001) cleaved-plane surface were selected based on the previous work.^34^ A 2 × 2 × 1 Å and 1 × 1 × 1 Å *k*-point sampling was used for MgO and Mg(OH)_2_ slabs, respectively.

All systems considered were totally optimized to reach atomic forces and total energies less than 0.05 eV Å^–1^ and 10^–4^ eV, respectively. A kinetic energy cutoff of 450 eV was set for all calculations. The van der Waals-corrected functional Becke88 optimization (optB88)^35^ was adopted for the consideration of noncovalent chemical interactions between molecules and clusters. The adsorption energy of the molecule is given by the following equation:^36^
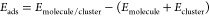
1where *E*_molecule/cluster_ is the total energy of the cluster with the adsorbed molecule, *E*_molecule_ is the total energy of the isolated molecule, and *E*_cluster_ is the total energy of the bare cluster. Under this definition, the negative adsorption energy indicates an exothermic and favorable process. The electrons gained or lost are defined as the difference of valence electrons of an atom in the adsorbed system from the atom in a free molecule or a substrate, according to the equation Δ*q* = *q*_after adsorption_ – *q*_before adsorption_. The negative and positive values indicate electrons gained and lost, respectively.

The charge transfer between the molecule and the cluster is given by the charge density difference (CDD) Δρ(*r*):
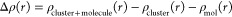
2where ρ_cluster+molecule_(*r*), ρ_cluster_, and ρ_mol_(*r*) are the charge densities of the cluster with the adsorbed molecule, the bare cluster, and the isolated molecule, respectively. The Bader analysis was used to calculate the charge transfer between the molecules and the clusters.^37^

The Arrhenius equation is given by the following formula:
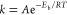
3where *k* is the rate constant, *A* is the pre-exponential factor, *E*_b_ is the activation energy or the energy barrier for a reaction, *R* is the universal gas constant, and *T* is the absolute temperature.^38^

The electron localization function (ELF) was calculated to obtain the distribution of electrons in the considered structures. The degree of charge localization in real space is depicted by the value of the ELF (between 0 and 1), where 0 represents a free electronic state and 1 represents a perfect localization. An isosurface value of 0.65 was adopted in this work.^39^

The climbing image–nudged elastic band (CI-NEB) method^40^ was used to obtain the reaction pathway of the molecule on the cluster. The AIMD simulations were performed at room temperature of 300 K. The simulation lasted for ∼5 ps with a time step of 1 fs, and the temperature was controlled by a Nose–Hoover thermostat.^41^

## Results and Discussion

### MgO Interaction with CO_2_ and H_2_O

**Figure 1:**
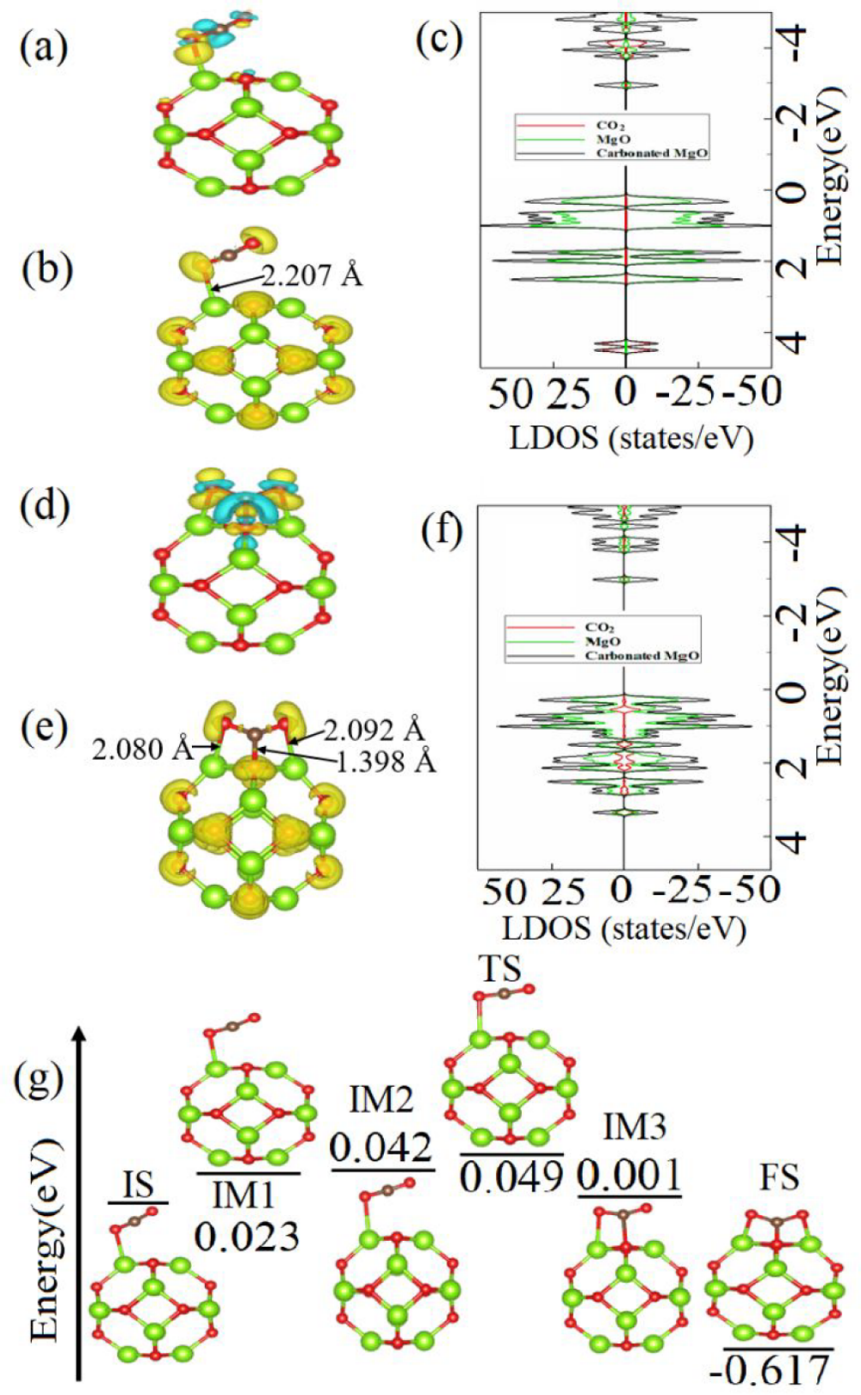
(a) Lowest-energy configuration of the CO_2_ molecule physisorbed on the MgO cluster combined with the CDD isosurface plot (0.003 Å^–3^). (b) ELF and (c) DOS and LDOS for the CO_2_–physisorbed MgO cluster. (d) Lowest-energy configuration of the CO_2_ molecule chemisorbed on the MgO cluster combined with the CDD isosurface plot (0.009 Å^–3^). (e) ELF and (f) DOS and LDOS for the CO_2_-chemisorbed MgO cluster. (g) Energy barrier and atomic structures corresponding to the minimum-energy pathway for the chemisorption process of the CO_2_ molecule on the MgO cluster.

The interaction of the MgO cluster with the CO_2_ molecule is considered to simulate the formation of MgO–CO_2_ (MgCO_3_) as the main precursor to HMCs. For this, various absorption configurations of the CO_2_ molecule on the MgO cluster are considered (more details see Figure S1 in the Supporting Information). Figure 1a shows the lowest-energy configuration structure of the CO_2_ molecule adsorbed on the MgO cluster combined with the CDD plot. In the most stable configuration, the O atom of the CO_2_ molecule is bonded to the Mg atom of the MgO cluster. The length of the created Mg–O bond is 2.207 Å. The length of the C–O bond of the CO_2_ molecule is elongated from 1.174 Å (bare CO_2_) to 1.188 Å (CO_2_ after adsorption on MgO). It is also found that the ∠(O–C–O) angle of CO_2_ adsorbed on the MgO cluster decreases to 171.94° compared to 179.95° for the bare CO_2_. Table S1 combines the results for the adsorption energy *E*_ads_ and charge transfer Δ*q* between the CO_2_ molecule and the MgO cluster. It is shown that *E*_ads_ of the CO_2_ molecule on the MgO cluster is −0.42 eV. According to the CDD plot (see Figure 1a), the CO_2_ molecule acts as an acceptor to the MgO cluster with the charge transfer from the surface to the molecule of 0.092 *e* (see Table S1), which can be attributed to the basicity of the MgO cluster, as it can donate a pair of nonbonding electrons following the Lewis base role.^21^ The observed elongation of the C–O bond and the enhanced charge transfer between the cluster and molecule suggest a strong interaction between them. The high electronegativity of O atoms of the molecule can be the driving force for the observed charge transfer compared to that of Mg atoms of the cluster. However, the ELF analysis (see Figure 1b) shows that electron density is mainly located at the Mg–O bond, which indicates electron depletion from the surface of the cluster to the CO_2_ molecule, and at the O atoms of the CO_2_ molecule, indicating that strong covalent bonding remains only within the molecule.

To deeper understand the interaction of the CO_2_ molecule with the MgO cluster, density of states (DOS) and local density of states (LDOS) analyses of CO_2_–adsorbed MgO are performed (see Figure 1c). The bare MgO cluster has higher HOMO and HOMO–1 states than the CO_2_ molecule, which indicates its tendency to oxidize the molecule, whereas the CO_2_ molecule possess LUMO and LUMO+1 states, which verifies its ability to gain electrons. Moreover, strong overlapping of LUMO and LUMO+1 states is observed upon the interaction between the molecule and the cluster, suggesting a strong interaction between them. In addition, AIMD simulations are conducted to study the interaction of the CO_2_ molecule with the MgO cluster at room temperature. The AIMD calculations (see Movie S1) confirm the possibility of the chemisorption of CO_2_ on the MgO cluster at room temperature and suggest a low energy barrier *E*_b_ for the reaction, as it is proposed from the *E*_a_ and charge transfer calculations. Therefore, the chemisorption process of CO_2_ on MgO is further considered.

The chemisorbed configuration of CO_2_ is chosen based on the AIMD-obtained configuration (see Figure S2). In that case, the length of the Mg–O bond formed between the cluster and the molecule is 2.080 Å, which is shorter than that in the physisorbed state (2.207 Å). The length of the newly formed Mg–O bond in the chemisorbed configuration is 2.092 Å. The C–O bond lengths of the CO_2_ molecule are 1.269 and 1.266 Å, which are significantly longer than those of the CO_2_ in its physisorbed state (1.188 Å). This indicates that C–O bonds of CO_2_ are highly elongated upon it interaction with Mg atoms. The ∠(O–C–O) angle of 179.95° of bare CO_2_ decreases to 129.69° for CO_2_ adsorbed on the MgO cluster. The CDD plot (see Figure 1d) and the Bader charge transfer analysis (see Table S1) predict that CO_2_ is an acceptor to MgO as it accumulates 0.117 *e* from the MgO cluster. The amount of charge transferred from MgO to chemisorbed CO_2_ is higher than that from MgO to physisorbed CO_2_ (see Table S1). Furthermore, *E*_ads_ of CO_2_ on MgO in its chemisorbed state is −1.05 eV (see Table S1), which is more than twice higher that of CO_2_ physisorbed on MgO. From Figure 1e, which shows ELF of CO_2_ chemisorbed on MgO, it is seen that electron localization located on the C–O bond formed between CO_2_ and MgO. In addition, strong electron redistribution is observed on O atoms of CO_2_, suggesting the formation of covalent bonds between the molecule and the cluster while the C–O covalent bonds of the CO_2_ molecule remain stable. That contribute to the depletion of electrons from the surface to the molecule as it is observed in the CCD plot in Figure 1d.

According to the DOS and LDOS plots in Figure 1f, there is a strong hybridization of the HOMO, HOMO–1, and LUMO+1 states of the MgO cluster and the CO_2_ molecule, indicating a strong interaction between them and signifying the possibility of chemisorption of the CO_2_ molecule on the MgO cluster. The AIMD simulations also suggest that the chemisorption of CO_2_ on MgO is favorable (see Movie S1 and Figure S2). Thus, the possible reaction mechanism for the transformation process for the CO_2_ molecule on the MgO cluster from physisorbed to chemisorbed state is further studied through the NEB approach. The energy profile and related atomic configurations for the initial state (IS), transition state (TS), intermediate states (IM), and final state (FS), showing the transition of the CO_2_ molecule from the physisorbed state to the chemisorbed state, are depicted in Figure 1g. TS with an energy level of 0.049 eV proposes the low energy barrier *E*_b_ for this transition (see Table S2). It seems that the O atom of CO_2_ has a high tendency to oxidize the Mg atom of the cluster. This oxidation is expedited at IM3 by the approach and further bonding of the C atom of the molecule to the O of the cluster, which leads to a drop of *E*_b_ to 0.001 eV. At FS, the second O atom of CO_2_ is bonded to the Mg atom of the cluster, and *E*_b_ further drops to −0.617 eV, which suggests the reaction is exothermic.

To summarize, the elongation of the C–O bond and the decrease of the ∠(O–C–O) angle of the CO_2_ molecule upon its chemisorption on the MgO cluster comparing to physisorption lead to an increase of *E*_a_.^24^ In addition, higher charge transfer from the cluster to the CO_2_ molecule during chemisorption stabilizes the adsorption of the CO_2_ molecule on the cluster.^42^ These results are well agreed with found low *E*_b_ of exothermic transition of CO_2_ from physisorbed state to chemisorbed state and with experimental observations confirming that the calcination of magnesite (MgCO_3_) is an endothermic process.^5^ Therefore, the chemisorption of CO_2_ on MgO occurs favorably under the reaction conditions.

**Figure 2:**
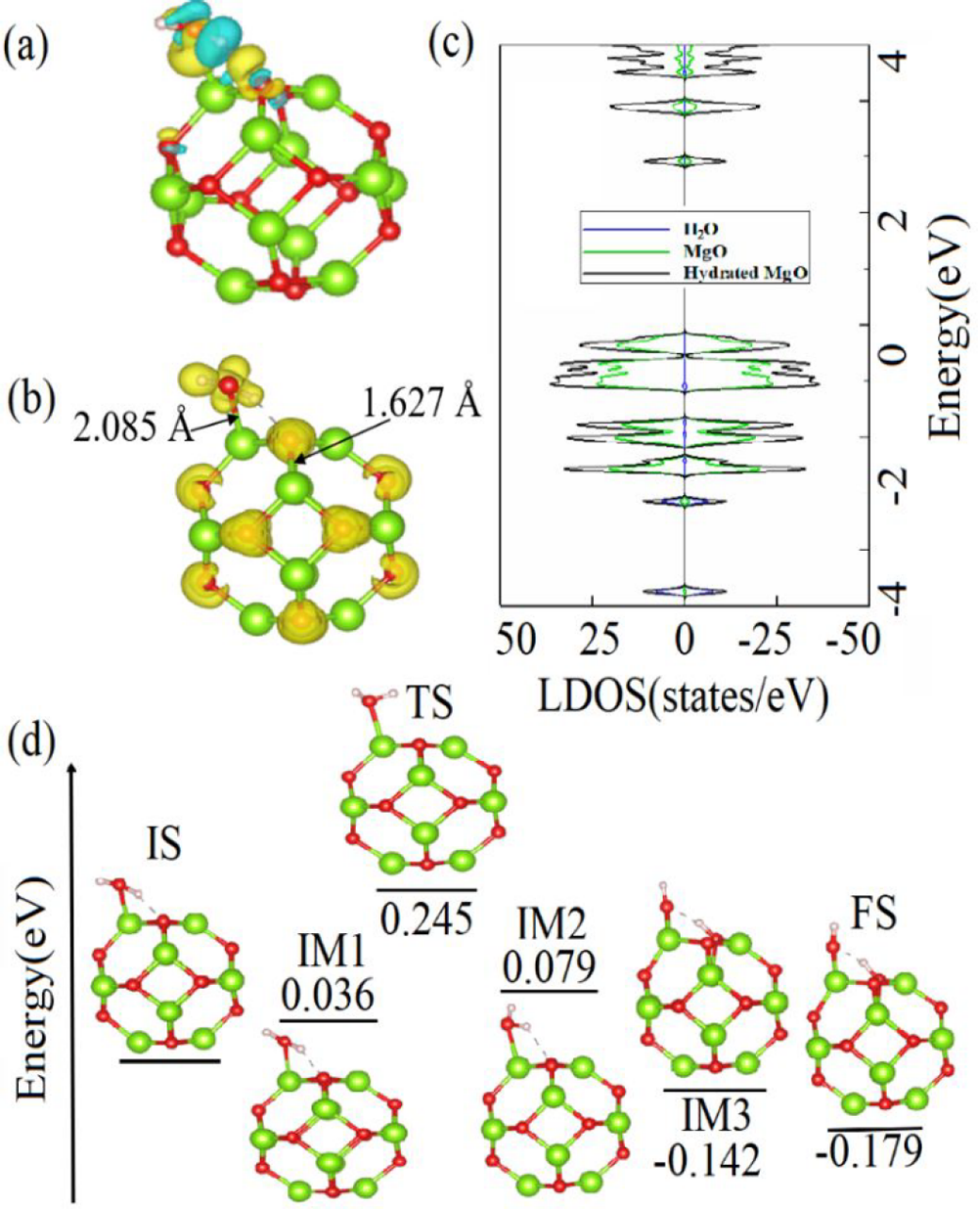
(a) Lowest-energy configuration of the H_2_O molecule on the MgO cluster combined with the CDD isosurface plot (0.003 Å^–3^). (b) ELF and (c) DOS and LDOS for the H_2_O–adsorbed MgO cluster. (d) Energy barrier and atomic structures corresponding to the minimum-energy pathway for the hydration of the MgO cluster.

The reaction of H_2_O with MgO leads to the formation of Mg(OH)_2_, a phase that might also undergo carbonation, which results in the HMC formation. Hence, the hydration of the MgO cluster is also investigated. All possible absorption configurations of H_2_O on the MgO cluster are considered (see Figure S3). According to Table S1, *E*_ads_ for the most energetically favorable configuration of adsorbed H_2_O on the MgO cluster (see Figure 2a) is −0.95 eV. In this configuration, the Mg–O bond between the O atom of the H_2_O molecule and the Mg atom of the MgO cluster and the H–O bond between the H atom of the molecule and the O atom of the cluster are formed. The length of the Mg–O and H–O bonds is found to be 2.085 and 1.627 Å, respectively. Moreover, the length of the H–O bond of the H_2_O molecule before bonding to the cluster is 0.936 Å, and it is elongated to 1.036 Å after adsorption, signifying the tendency of H_2_O to bind to MgO. According to the Bader charge transfer analysis and the CCD plot (see Figure 2a), the H_2_O molecule is a strong electron acceptor to the cluster with Δ*q* = −1.122 *e* (see Table S1). The basicity of the MgO cluster facilitates the electrons transfer from the O atom of the cluster to the H_2_O molecule, while a higher electronegativity of the O atom of the molecule facilitates electron depletion toward H atoms. Such significant charge redistribution between the MgO cluster and the H_2_O contributes to its adsorption.^36^

The ELF plot in Figure 2b shows the electron localization between the O atom of the H_2_O molecule and the Mg atom of the MgO cluster as well as the localization between the H atom of the H_2_O molecule and the O atom of the MgO cluster, which confirms electron depletions at these sites and suggests the formation of the H–O and Mg–O bonds between the molecule and the cluster. The DOS and LDOS plots in Figure 2c display the hybridization of H_2_O and MgO states at −3.3 and 4.9 eV and a weak interaction at 4.4 eV. The conducted AIMD simulation also confirms the dissociation of the H_2_O molecule on the MgO cluster and formation of the H–O and Mg–O bonds (see Movie S2 and Figure S4).

NEB calculations are performed to show the possible reaction mechanism of the H_2_O molecule dissociation on the MgO cluster. Figure 2d presents the energy profile and related atomic configurations for the IS, TS, IMs, and FS showing the dissociation of the H_2_O molecule of the MgO cluster. As it is seen, between IS and TS the H_2_O molecule bonds to the MgO cluster through the rotation of the H atom of the molecule (IM1). *E*_b_ of the H_2_O molecule dissociation on the MgO cluster at TS is found to be as high as 0.245 eV (see Table S2). Further reaction at IM2 and IM3 leads to the bonding of the H atom of the H_2_O molecule to the nearest O atom of the cluster and the consequent H_2_O dissociation at FS occurring with an energy release of 0.179 eV. A higher energy release during the carbonation (−0.617 eV) of the MgO cluster compared to that during hydration (−0.179 eV) of the MgO cluster indicates that the carbonation of the MgO cluster is a more exothermic process than its hydration. Therefore, the carbonated MgO is more thermodynamically stable. However, *E*_b_ for carbonation of the MgO cluster is 0.235 eV, which is lower than *E*_b_ of 0.245 eV for hydration of the MgO cluster. On the other hand, AIMD simulations suggest that the hydration of the MgO cluster passes faster than its carbonation (see Figure S4). Therefore, hydration and carbonation rates of the MgO cluster are compared based on the Arrhenius equation (eq 3), according to which the reaction rate depends on two factors: activation energy of the reaction and pre-exponential factor *A*. Therefore, besides the calculated *E*_b_, the *A* factor, describing the frequency of collisions between reactant molecules at a standard concentration, should be taken into consideration for the comparison of hydration and carbonation rates of the MgO cluster. The hydrolysis of the MgO cluster changes its structure due to a break of Mg–O bonds of the MgO cluster upon interaction with H_2_O, while the carbonation of the MgO cluster does not cause the alteration of the MgO cluster. This leads to a significant difference in the *A* factor for the hydration and carbonation of the cluster. As a result, the hydration of the MgO cluster is faster than its carbonation as it is shown by AIMD simulations (see Figure S4, Movie S1, and Movie S2). This observation is also in line with the fact that *E*_ads_ of the H_2_O molecule (−0.95 eV) on the MgO cluster is more than 2 times lower than that of the CO_2_ molecule (−0.42 eV) on the MgO cluster, which leads to faster hydration reaction. Faster hydration of the MgO cluster is also observed in AIMD simulations (see Figure S4) where the adsorption of the H_2_O molecule of the MgO cluster occurs ∼3 times faster than that of the CO_2_ molecule. Furthermore, to compare the hydration and the carbonation rate of the MgO cluster, AIMD simulations are performed to simulate a CO_2_- and H_2_O-saturated environment, consisting of three CO_2_ and three H_2_O molecules (see Movie S3). The trajectory of these molecules shows that hydration of MgO is significantly faster than its carbonation (see Figure S5), as all three considered H_2_O molecules bond to the MgO cluster before any of the CO_2_ molecules.

In summary, the formation of the H–O and Mg–O bonds between the H_2_O molecule and the MgO cluster verifies H_2_O chemisorption on the cluster. The calculated NEB energy profile diagram predicts that the H_2_O molecule dissociation on the MgO cluster is an exothermic process, and the carbonation of MgO is thermodynamically more favorable than its hydration. However, although the calculated *E*_b_ for the hydration of the MgO cluster is higher than that for its carbonation, the hydration of the MgO cluster is found to be faster, as confirmed by the calculated *E*_ads_ and AIMD simulations.

**Figure 3:**
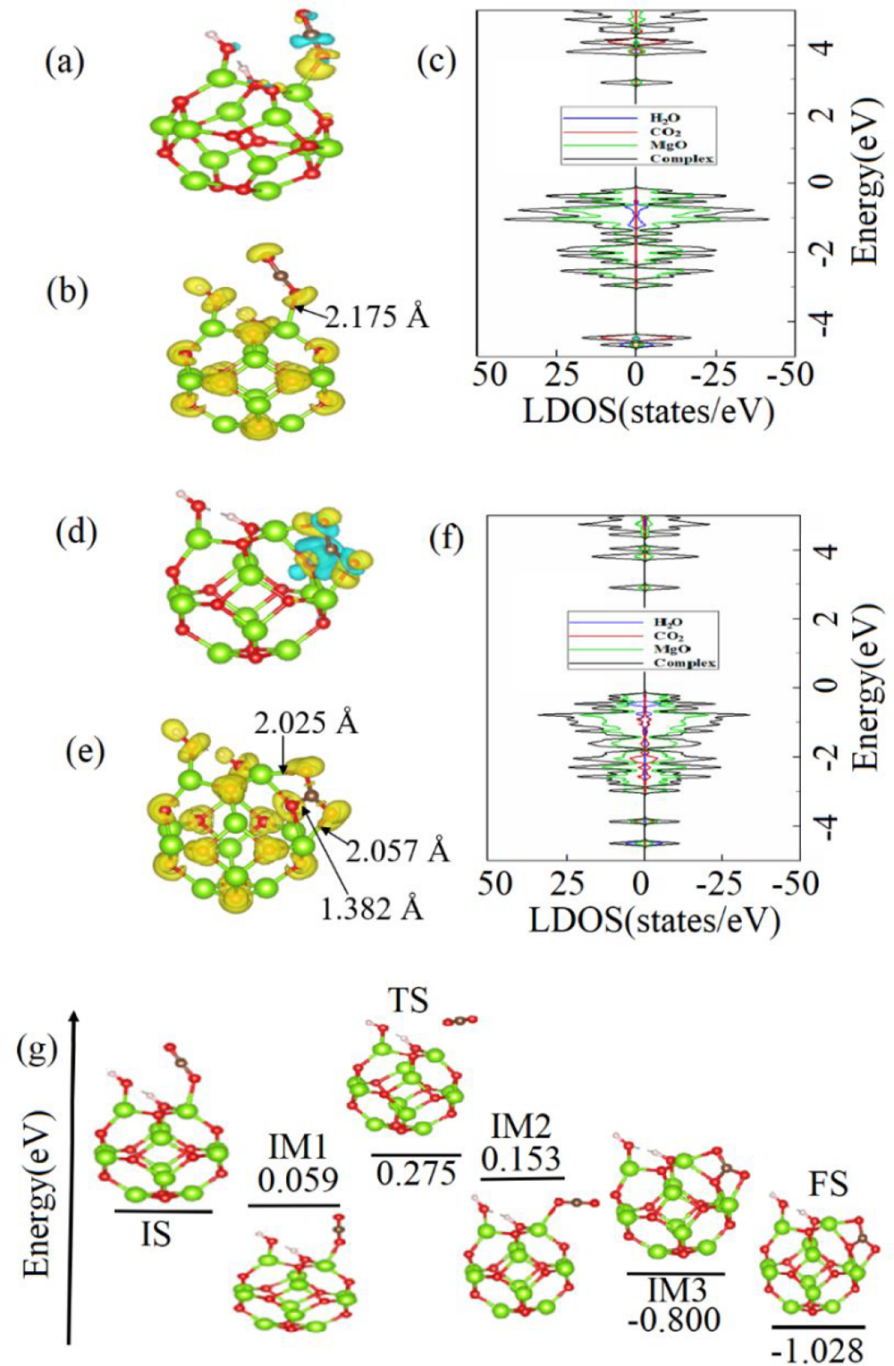
(a) Lowest-energy configuration of the physisorbed CO_2_ molecule on the hydrated MgO cluster combined with the CDD isosurface plot (0.003 Å^–3^). (b) ELF and (c) DOS and LDOS for the CO_2_–physisorbed hydrated MgO cluster. (d) Lowest-energy configuration of the chemisorbed CO_2_ molecule on the hydrated MgO cluster combined with the CDD isosurface plot (0.009 Å^–3^). (e) ELF and (f) DOS and LDOS for the CO_2_–chemisorbed hydrated MgO cluster. (g) Energy barrier and atomic structures corresponding to the minimum-energy pathway for the transition of the CO_2_ molecule from physisorbed to chemisorbed states on the hydrated MgO cluster.

As it is found that hydration of MgO occurs faster than its carbonation, the CO_2_ molecule interaction with the hydrated MgO cluster (previously found lowest-energy configuration of hydrated MgO is used) is studied. Several possible configurations of the CO_2_ molecule on the hydrated MgO cluster are considered (see Figure S6). Figure 3a shows the lowest-energy configuration of the CO_2_ molecule on the hydrated MgO cluster, where the O atom of the CO_2_ molecule is bonded to the Mg atom of the MgO cluster. The newly formed Mg–O bond has a length of 2.175 Å. The length of the C–O bond (the one closest to the cluster) of the adsorbed CO_2_ is elongated to 1.182 Å compared to that of bare CO_2_ of 1.174 Å, while another C–O bond of CO_2_ shortens to 1.165 Å. The ∠(O–C–O) angle of the CO_2_ molecule also decreases from 179.95° to 174.43° upon its adsorption.

The CDD plot in Figure 3a shows that the CO_2_ molecule is an acceptor to the hydrated MgO cluster as there is a depletion of the electron on the Mg atom of the cluster and accumulation of electrons on the O atom of the CO_2_ molecule. The Bader charge transfer analysis predicts that the amount of the charge transferred from the cluster to the molecule is 0.058 *e*. Importantly, *E*_ads_ of the CO_2_ molecule on the hydrated MgO cluster is −0.53 eV, which is lower than that of the CO_2_ molecule on the bare MgO cluster. This suggests stronger bonding of the CO_2_ molecule with the hydrated MgO cluster compared to the bare MgO cluster. The ELF plot in Figure 3b demonstrates the electron localization between the O atom of the molecule and the Mg atom of the cluster. It verifies the accumulation of electrons on the O atoms of the CO_2_ molecule and suggests that the C–O bonds of the molecule remain covalent. The DOS and LDOS plots in Figure 3c show strong overlapping of HOMO states of the H_2_O molecule and the MgO cluster in the range from −4.2 to −4.8 eV and strong overlapping of LUMO+1 states of the H_2_O molecule and the MgO cluster in the range from 3.4 to 4.5 eV, which confirms a strong bonding between the CO_2_ molecule and the hydrated MgO cluster. The conducted AIMD calculations predict the possibility of chemisorption of the CO_2_ molecule on the hydrated MgO cluster at room temperature. In the chemisorbed state, the O atoms of the CO_2_ molecule are bonded to the Mg atoms of the hydrated MgO cluster and the C atom of the CO_2_ molecule is bonded to the O atom of the hydrated MgO cluster (see Movie S4 and Figure S7). It is observed that the carbonation of bare MgO occurs slower than the carbonation of hydrated MgO due to the formation of OH groups on the MgO cluster during its hydration, which hinder the carbonation process.

To gain insights into the carbonation mechanism of hydrated MgO, the chemisorption process of CO_2_ on it is considered. The lowest-energy configuration of chemisorbed CO_2_ molecule on the hydrated MgO cluster (for more details see Figure S7) is shown in Figure 3d. Here, both O atoms of the CO_2_ molecule form chemical bonds with the Mg atoms of the hydrated MgO cluster. The C–O bonds of the CO_2_ molecule are elongated to 1.269 and 1.275 Å (compared to 1.174 Å of the bare CO_2_ molecule) upon its adsorption on the hydrated MgO cluster. The length of newly formed Mg–O bonds is 2.057 and 2.025 Å, while the length of the C–O bond formed between the C atom of the molecule and the O atom of the cluster is 1.382 Å. The ∠(O–C–O) angle of the adsorbed CO_2_ molecule is found to be 129.16°, which is lower than that of the CO_2_ molecule in its physisorbed state. The CDD plot in Figure 3d shows that the charge is mostly distributed on the CO_2_ molecule and partially on the O atom of the MgO cluster bonded to the C atom of the CO_2_ molecule. The basicity of the hydrated MgO cluster drives the electron transfer from the molecule to the hydrated cluster. According to the Bader charge transfer analysis, the chemisorbed CO_2_ molecule gains 0.086 *e* from the hydrated MgO cluster. Therefore, the amount of the charge transferred from the hydrated MgO cluster to the chemisorbed CO_2_ molecule is higher than that from the hydrated MgO cluster to the physisorbed CO_2_ molecule (see Table S1). The calculated *E*_ads_ of −1.55 eV for the CO_2_ molecule chemisorbed on the hydrated MgO cluster is higher than that of the CO_2_ molecule chemisorbed on the bare MgO cluster (−1.05 eV). The ELF plot in Figure 3e depicts electron localizations between the O atoms of the chemisorbed CO_2_ molecule and the Mg atoms of the hydrated MgO cluster and the C atom of the chemisorbed CO_2_ molecule and the O atom of the hydrated MgO cluster, which suggests the existence of the covalent Mg–O and C–O bonds between the cluster and the molecule. Meanwhile, the covalent bonding between the H_2_O molecule and the MgO cluster remains unchanged. According to DOS and LDOS plots presented in Figure 3f, there is a strong hybridization of HOMO and HOMO–1 states of the hydrated MgO cluster and the chemisorbed CO_2_ molecule. The overlapping of the cluster and the molecule is also observed at −3.8, −4.5, and 4.6 eV.

An *E*_b_ of 0.275 eV (see Table S2) for the transition of the CO_2_ molecule from the physisorbed state to the chemisorbed state on the hydrated MgO cluster is calculated by the NEB approach (see Figure 3g). The transition involves the IM2 stage, where the O atom of the CO_2_ molecule oxidizes the Mg atom of the MgO cluster, which leads to the drop of *E*_b_ to 0.153 eV. This triggers an exothermic process of bonding the C and O atoms of the CO_2_ molecule to the hydrated MgO cluster at the FS state via the IM3 (−0.800) state with the released energy of 1.028 eV. According to calculated reaction energies in the carbonation process of bare MgO (−0.617 eV) and hydrated MgO (−1.028 eV), carbonation of the hydrated MgO is thermodynamically more favorable. However, *E*_b_ for the transition of CO_2_ from the physisorbed state to the chemisorbed state on hydrated MgO (0.275 eV) is higher than that of CO_2_ on bare MgO (0.234 eV). Therefore, CO_2_ chemisorption on hydrated MgO is kinetically unfavorable. This matches the AIMD simulation results (see Figures S2 and S6), where the carbonation of bare MgO is faster than that of the hydrated MgO. Importantly, this verifies the fact that the initial hydration of MgO can hinder its carbonation.^15^

In summary, the chemisorption of the CO_2_ on the MgO cluster is found to be the most energetically favorable. The charge redistribution between the MgO cluster and the CO_2_ molecule during the chemisorption^37,38^ and the comparison of the energy released during carbonation of the bare and the hydrated MgO clusters suggest carbonation of the bare MgO cluster is faster than that of the hydrated MgO cluster, which uncovers the hindrance effect of H_2_O on the carbonation of MgO. The observed results are also supported by AIMD simulations (see Movie S4 and Figure S7).

### Mg(OH)_2_ Interaction with CO_2_ and H_2_O

**Figure 4:**
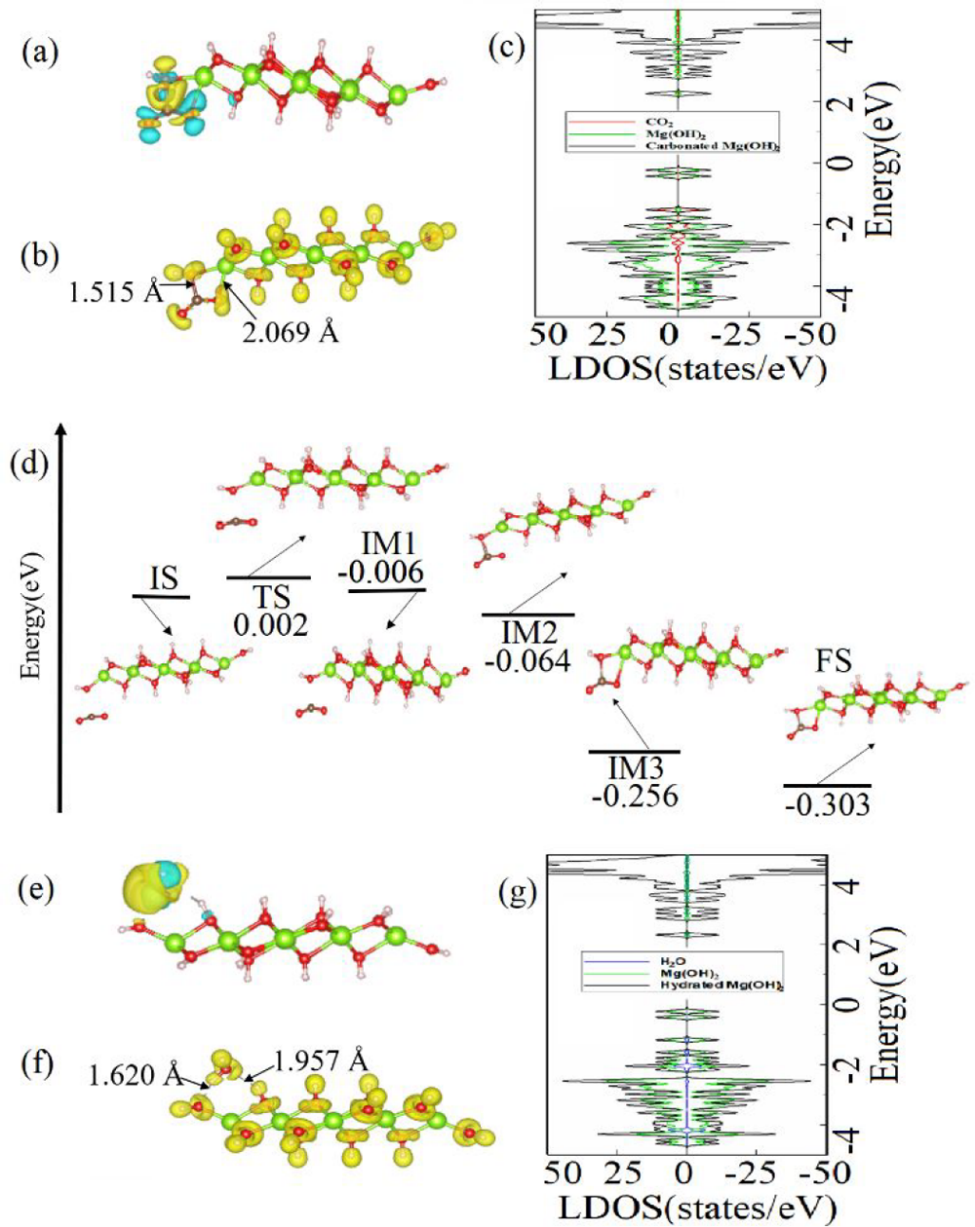
(a) Atomic structure of the lowest-energy configuration of the CO_2_ molecule on the Mg(OH)_2_ cluster combined with the CDD isosurface plot (0.006 Å^–3^). (b) ELF and (c) DOS and LDOS for the CO_2_–adsorbed Mg(OH)_2_ cluster. (d) Energy barrier and atomic structures corresponding to the minimum-energy pathway for the carbonation of the Mg(OH)_2_ cluster. (e) Atomic structure of the lowest-energy configuration of the H_2_O molecule on the Mg(OH)_2_ cluster combined with the CDD isosurface plot (0.009 Å^–3^). (f) ELF and (g) DOS and LDOS for the H_2_O–adsorbed Mg(OH)_2_ cluster.

In RMC reactions, the carbonation of Mg(OH)_2_ leads to the production of a range of HMCs.^12,13^ Therefore, the mechanism of the carbonation of Mg(OH)_2_ is further studied. For that, several possible configurations of the CO_2_ molecule and the Mg(OH)_2_ cluster are examined (see Figure S8). The most favorable sites for the CO_2_ molecule adsorption on Mg(OH)_2_ are located at its edges. Figure 4a combines the atomic structure of the lowest-energy configuration and the CDD plot for the CO_2_ molecule adsorbed on the Mg(OH)_2_ cluster. In that case, the C atom of the CO_2_ molecule is located below the O atom at the edge of the Mg(OH)_2_ cluster and is bonded to the O atom of the cluster. In the same way, the O atom of the CO_2_ molecule is bonded to the Mg atom at the edge of the Mg(OH)_2_ cluster and forms the Mg–O bond of length 2.069 Å. Upon adsorption, the C–O bonds in the CO_2_ molecule elongates from 1.174 Å (bare CO_2_) to 1.266 Å, while a newly formed C–O bond between the CO_2_ molecule and Mg(OH)_2_ has a length of 1.515 Å and ∠(O–C–O) changes from 179.95° to 136.88°. The CDD plot in Figure 4a displays the charge transfer from O atoms at the edge of Mg(OH)_2_ to the CO_2_ molecule. The Bader charge transfer analysis suggests that CO_2_ acts as an acceptor to the Mg(OH)_2_ cluster, with the charge transfer from the cluster to the molecule of 0.397*e* (see Table S1). This verifies the Lewis basicity of the Mg(OH)_2_ cluster. According to Table S1, the *E*_ads_ of CO_2_ on Mg(OH)_2_ is −0.69 eV.

The ELF plot in Figure 4b shows electron localization between the O atom of the CO_2_ molecule and the Mg atom of the Mg(OH)_2_ cluster, which characterizes electron transfer and strong bonding between the molecule and the edge of the cluster. The covalent bonding within the molecule also remains stable, as predicted by the charge localization on both the C–O bonds of the CO_2_ molecule. The DOS and LDOS plots for the CO_2_–adsorbed Mg(OH)_2_ cluster are shown in Figure 4c. The observed strong orbital hybridization of CO_2_ and Mg(OH)_2_ at the energy of −1.7 eV and in a range from −2 to −3.7 eV confirms the strong interaction between CO_2_ and Mg(OH)_2_ proposed by the charge transfer and ELF analysis. Figure 4d depicts the potential energy profile and atomic structures corresponding to the minimum-energy pathway for the carbonation of the Mg(OH)_2_ cluster. It is shown that *E*_b_ for the carbonation of Mg(OH)_2_ is as low as 0.002 eV (TS in Figure 4d), which is equivalent to a spontaneous process at room temperature. To reach the chemisorbed state at FS (−0.303 eV), the CO_2_ molecule passes through the IM2 state (−0.064 eV), where the C atom of the molecule bonds to the O atom of the cluster, and IM3 (−0.256), at which point the O atom of the molecule forms a bond with the Mg atom of the cluster. It is also noted that the carbonation of Mg(OH)_2_ is a highly exothermic process.

In summary, the elongation of C–O bonds and the decrease of ∠(O–C–O) of the CO_2_ molecule, along with the strong charge transfer between the molecule and the Mg(OH)_2_ cluster, play a dominant role in CO_2_ chemisorption on Mg(OH)_2_. Despite the chemisorption of CO_2_ on Mg(OH)_2_ occurring only at the edges, the chemisorption mechanism of CO_2_ for Mg(OH)_2_ is similar to that for MgO. In both cases, chemisorption of CO_2_ is an exothermic process with low *E*_b_ and significant energy release. However, the activation energy for the carbonation of Mg(OH)_2_ (0.002 eV) is significantly lower than that for MgO (0.049 eV), confirming that carbonation of the Mg(OH)_2_ is faster than that of the MgO. In turn, the energy released during the MgO carbonation (−0.617 eV) is about 2 times lower than that during the Mg(OH)_2_ carbonation (−0.303 eV), which suggests that the carbonation of the MgO cluster is more thermodynamically favorable.

Mg(OH)_2_ is often affected by aqueous environments; therefore, the interaction of the H_2_O molecule with the Mg(OH)_2_ cluster can play a key role in HMC formation. From the studied configurations for that interaction of H_2_O with Mg(OH)_2_ (see Figure S9), the lowest-energy configuration is related to the H_2_O molecule located at the edge of the Mg(OH)_2_ cluster. The length of the O–H bond of the bare H_2_O molecule (1.972 Å) is shortened to 1.020 Å upon the H_2_O molecule bonding to the Mg(OH)_2_ cluster, while the O–H bond at the edge of the Mg(OH)_2_ cluster is elongated from 0.965 to 0.984 Å. The CDD plot in Figure 4e shows that the charge is mostly distributed on the H_2_O molecule and partially at the edge of the Mg(OH)_2_ cluster. The Bader charge transfer analysis predicts the H_2_O molecule to be a weak acceptor to the Mg(OH)_2_ cluster which accumulates 0.044*e* (see Table S1). *E*_ads_ of the H_2_O molecule on the Mg(OH)_2_ cluster is −0.74 eV (see Table S1). The ELF plot in Figure 4f shows insignificant electron distributions between the O atom of the H_2_O molecule and the H atom of the Mg(OH)_2_ cluster. Meanwhile, low electron density between the H atom of the H_2_O molecule and the O atom of the Mg(OH)_2_ cluster indicates weak interaction between them. Moreover, orbital localization between the O–H bonds of the H_2_O molecule shows that the covalent bonds of the molecule remain stable. The DOS and LDOS plots for the H_2_O molecule adsorbed on the Mg(OH)_2_ cluster, shown in Figure 4g, also suggest a weak interaction between the molecule and the cluster at −1.5, −1.8, and −2 eV and in the ranges from −2.2 to −2.5 eV and from −4.1 to −4.2 eV.

In summary, it is found that the H_2_O molecule is located at the edges of the Mg(OH)_2_ cluster. The calculated low *E*_ads_ and weak charge transfer between the H_2_O molecule and the Mg(OH)_2_ cluster suggest that H_2_O is physisorbed on Mg(OH)_2_. However, it is well-known that the presence of H_2_O facilitates the formation of HMCs in accessible pores during the carbonation process.^37^

**Figure 5:**
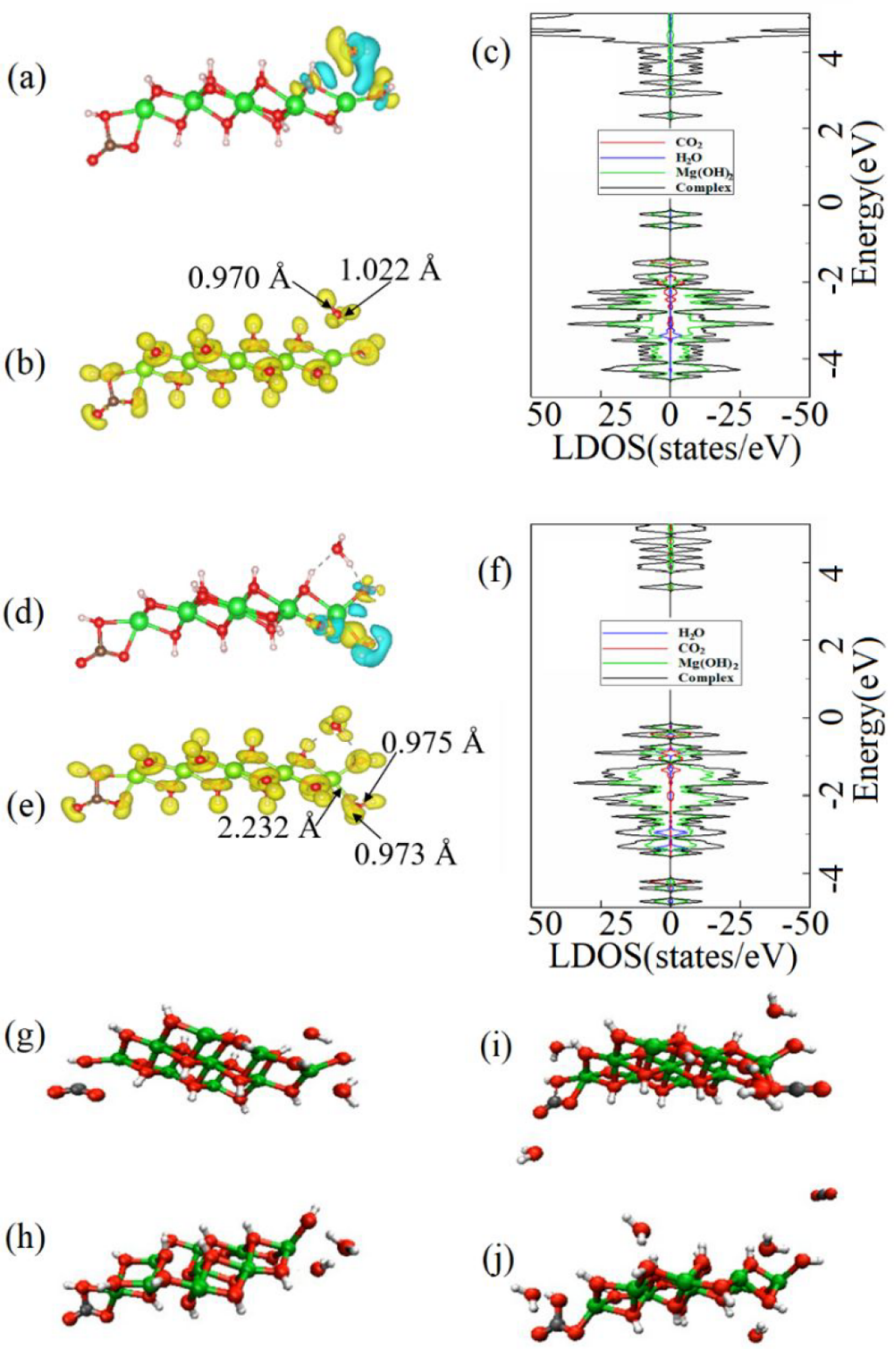
(a) Lowest-energy configuration of the H_2_O molecule on the carbonated Mg(OH)_2_ cluster with the CDD isosurface plot (0.001 Å^–3^). (b) ELF and (c) DOS and LDOS for H_2_O molecules on the carbonated Mg(OH)_2_ cluster. (d) Lowest-energy structure of the H_2_O molecules on the carbonated Mg(OH)_2_ cluster combined with the CDD plot (0.001 Å^–3^). (e) ELF and (f) DOS and LDOS for the H_2_O molecule on the carbonated Mg(OH)_2_ cluster. (g) Initial and (h) final (chemisorbed state) configurations of CO_2_ and H_2_O molecules on the Mg(OH)_2_ cluster. (i) Initial and (h) final configurations of CO_2_ and H_2_O molecules on the carbonated Mg(OH)_2_ cluster.

To investigate a mechanism of reaction of nesquehonite formation (Mg(OH)_2_ + CO_2_ + 2H_2_O → MgCO_3_·3H_2_O), at the first step, the simultaneous interaction of the carbonated Mg(OH)_2_ cluster and the H_2_O molecule (Mg(OH)_2_ + CO_2_ + H_2_O) is considered. At the second step, one more H_2_O molecule is introduced to the system studied at the first step (CO_2_ + 2H_2_O + Mg(OH)_2_). Although the natural process of the nesquehonite formation also includes nucleation and growth from species in solution, the studied reaction will still help to understand possible nucleation or growth paths of nesquehonite. At the first step, various configurations of one H_2_O molecule (see Figure S10) on the carbonated Mg(OH)_2_ cluster are considered. At the lowest-energy configuration, the H_2_O molecule is bonded to the edge of the Mg(OH)_2_ cluster. The length of the O–H bonds increases to 0.970 and 1.022 Å compared to these of the bare H_2_O molecule (0.936 Å). The distance between the H atom of the H_2_O molecule and the O atom of the Mg(OH)_2_ cluster is 1.620 Å, while the distance between the O atom of the H_2_O and the H atom of the Mg(OH)_2_ cluster is 1.957 Å. According to Table S1, *E*_ads_ of the H_2_O molecule on the Mg(OH)_2_ cluster is −0.86 eV. The CDD plot in Figure 5a shows that there is a depletion of electrons at the edge O atoms of the Mg(OH)_2_ cluster and charge accumulation at the H atoms of the H_2_O molecule. The Bader charge transfer analysis shows that the H_2_O molecule gains 0.046*e* from the Mg(OH)_2_ cluster, which confirms that H_2_O is a weak acceptor to Mg(OH)_2_ (see Table S1). In addition, the ELF plot in Figure 5b shows H_2_O is physisorbed on Mg(OH)_2_, as there is no electron density localization between the Mg(OH)_2_ cluster and the H_2_O molecule, while the H–O bonds of H_2_O retain their covalent nature. Figure 5c represents the DOS and LDOS plots for the H_2_O molecule adsorption on the carbonated Mg(OH)_2_ cluster. A small overlapping of states of the H_2_O molecule and the carbonated Mg(OH)_2_ cluster is observed in the range from −3.0 to −3.7 eV, verifying the weak interaction between them.

In addition, at the second step, the second H_2_O molecule is introduced to the Mg(OH)_2_ + CO_2_ + H_2_O system obtained at the first step (see Figure S11). The lowest-energy configuration of the H_2_O molecule on the Mg(OH)_2_ + CO_2_ + H_2_O system is shown in Figure 5d, where the second H_2_O molecule is also located at the edge of the carbonated Mg(OH)_2_ cluster. The length of the H–O bonds of the bare H_2_O molecule is 0.971 Å, while they slightly elongate to 0.975 and 0.973 Å after adsorption. The distance between the O atom of the H_2_O molecule and the Mg atom of the Mg(OH)_2_ cluster is 2.232 Å. According to Table S1, *E*_ads_ of the H_2_O molecule on the Mg(OH)_2_ + CO_2_ + 2H_2_O system is −0.46 eV. The CDD plot in Figure 5d displays the depletion of electrons at O atoms located at the edge of the Mg(OH)_2_ cluster and charge accumulation at the H atoms of the H_2_O molecule. According to the Bader charge transfer analysis (see Table S1), the H_2_O molecule is a weak acceptor to the Mg(OH)_2_ cluster with the charge transfer of 0.037*e* from the cluster to the molecule. The ELF plot in Figure 5e shows no election density localization between the second H_2_O molecule and the Mg(OH)_2_ + CO_2_ + H_2_O system, which means there is a weak interaction between them. The DOS and LDOS plots in Figure 5f also display a weak interaction of the H_2_O molecule and the cluster at the range from −2.8 to −3.5 eV.

AIMD simulations are used to investigate the reaction for the formation of HMCs via the interaction of the CO_2_ and H_2_O molecules with the Mg(OH)_2_ cluster (see Movie S5 and Figure 5g,h). As it is shown, the CO_2_ and H_2_O molecules are bonded at the edges of the Mg(OH)_2_ cluster, which suggests that the formation of HMCs starts at the edges of Mg(OH)_2_. In addition, AIMD simulations are conducted to consider the effect of large amount of H_2_O and CO_2_ molecules on the formation of HMCs (see Movie S6). For that, two H_2_O and one CO_2_ molecules are added to the previously considered Mg(OH)_2_ + CO_2_ + 2H_2_O system. As shown in Figure 5h, the first CO_2_ molecule can carbonate the Mg(OH)_2_ cluster. However, after the bonding of the first CO_2_ molecule to the cluster, the second CO_2_ molecule is unable to bind to the carbonated Mg(OH)_2_ cluster (Figure 5i,j). This suggests that the formation of an early layer of carbonates in RMC-based concrete formulations may limit the continuation of carbonation by forming a physical barrier that prohibits further interaction between Mg(OH)_2_ and CO_2_. These limitations in carbonation of Mg(OH)_2_ can cause large amounts of unreacted crystals leading to relatively low strength and porous microstructures.^15,43^ Although the presence of H_2_O molecules provides the medium for carbonation and further transformation of Mg(OH)_2_ into HMCs and is required for the continuous formation of HMCs,^44^ according to the AIMD simulations, excessive H_2_O hinders CO_2_ penetration to the Mg(OH)_2_ surface. Therefore, to maintain CO_2_ diffusion for carbonation of Mg(OH)_2,_ the amount of H_2_O should be properly controlled.^40^ The predicted results of Mg(OH)_2_ passivation with the formation of the barrier of carbonates and H_2_O hindrance effect on carbonation of MgO correspond to the carbonation mechanisms of portlandite.^45^

**Figure 6:**
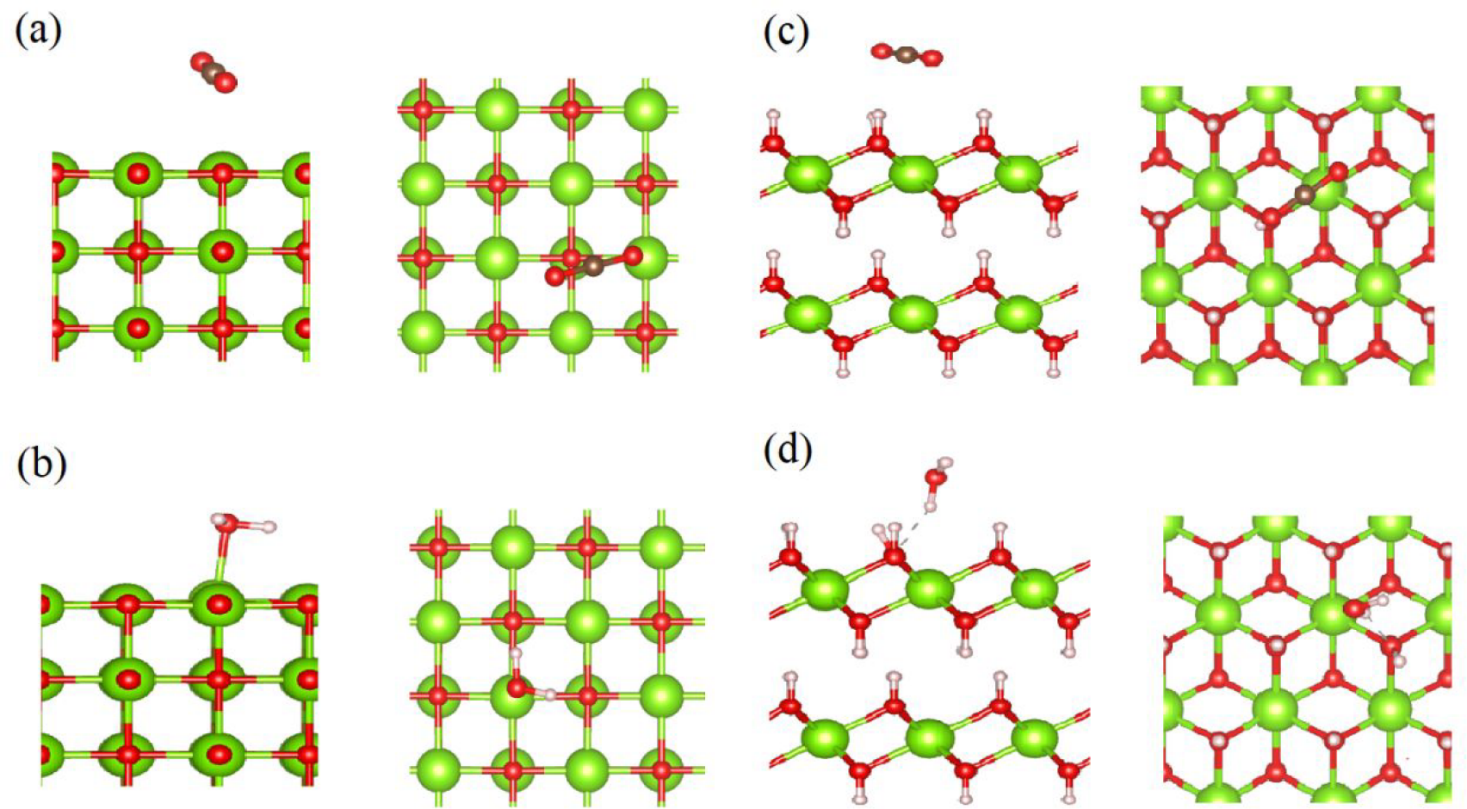
Side and top views of the lowest-energy configuration of (a) CO_2_ and (b) H_2_O molecules on the MgO(001) surface. Side and top views of the lowest-energy configuration of (c) CO_2_ and (d) H_2_O molecules on the Mg(OH)_2_(001) surface.

As the adsorption behavior of molecules on the clusters may be different from that on the bulk materials. In addition, the interaction of the CO_2_ and H_2_O molecules with clusters and with the bulk MgO and Mg(OH)_2_ is compared. Figures 6a and 6b represent the lowest-energy configuration of the CO_2_ and H_2_O molecules on MgO(001). Figure 6a shows the O atom of CO_2_ molecule is located above Mg atom of MgO(001). The length of C–O bonds in the CO_2_ molecule elongates from 1.174 Å (bare CO_2_) to 1.182 and 1.175 Å, and ∠(O–C–O) decreases from 179.95° to 176.81°. The calculated *E*_ads_ for CO_2_ on the MgO(001) surface is −0.34 eV (see Table S3). This indicates weak adsorption of CO_2_ on the bulk MgO(001) compared to the physisorbed CO_2_ on the MgO cluster (*E*_ads_ = −0.42 eV). According to Figure 6b, the O atom of the H_2_O molecule is located above the Mg atom of MgO(001). The length of the H–O bonds of the H_2_O molecule elongates from 0.972 Å (bare H_2_O) to 0.983 and 0.977 Å. *E*_ads_ = −0.58 eV of the H_2_O molecule on MgO(001) (see Table S3) is found to be lower than that of the H_2_O molecule (−0.95 eV) on the MgO cluster. The length of the Mg–O bond formed between the O atom of the H_2_O molecule and the Mg atom at the MgO(001) surface is 2.239 Å, which is longer than the Mg–O bond formed between the O atom of the H_2_O molecule and the Mg atom of the MgO cluster (2.085 Å). Therefore, for both H_2_O and CO_2_ molecules their adsorption and possible dissociation at the edges or defective surfaces of the MgO crystal, presented here via clusters, are more favorable. This result well matches the previously reported observation on weak adsorption of the CO_2_ molecule on the MgO(001) surface.^24^

Figures 6c and 6d show the lowest-energy configurations of the CO_2_ and H_2_O molecules on the Mg(OH)_2_(001) surface. Figure 6c indicates the location of the C atom of the CO_2_ molecule is located above the Mg–O bond of the Mg(OH)_2_(001) surface. The C–O bonds in the CO_2_ molecule elongates from 1.174 Å (bare CO_2_) to 1.177 and 1.178 Å, and ∠(O–C–O) changes from 179.95° to 179.00°. *E*_ads_ of the CO_2_ molecule on the Mg(OH)_2_(001) surface is as low as −0.25 eV, which is significantly lower than that of the CO_2_ molecule on the Mg(OH)_2_ cluster (−0.69 eV) (see Table S3). Figure 6d depicts the interaction of the H atom of H_2_O molecule with the O atom of the Mg(OH)_2_(001) surface. The length of the H–O bond of the H_2_O molecule near the surface elongates from 0.972 Å (bare H_2_O) to 0.994 Å, while another O–H bond is shortened to 0.952 Å. The *E*_ads_ of H_2_O molecule on the Mg(OH)_2_(001) surface is found to be −0.37 eV, which is lower than that of the H_2_O molecule on the Mg(OH)_2_ cluster (−0.74 eV) (see Table S3). In addition, the distance between the H_2_O molecule and the Mg(OH)_2_(001) surface of 2.017 Å is longer than that between the H_2_O molecule and the Mg(OH)_2_ cluster (1.957 Å). Similar to the case of MgO, the lower *E*_ads_ of the CO_2_ and H_2_O molecules on the Mg(OH)_2_ cluster, compared to that on the Mg(OH)_2_(001) surface, suggests stronger interaction of these molecules with the edge and/or defect-containing surface of the Mg(OH)_2_ crystal.

## Conclusions

In this study, the mechanism of potential reactions on MgO and Mg(OH)_2_ during HMC synthesis is investigated by DFT-based calculations. The results show that despite the energy barrier for the CO_2_ molecule adsorption on MgO is lower than that for the H_2_O molecule adsorption on MgO, the hydration of MgO is faster due to the difference in the frequency of CO_2_ and H_2_O molecules collisions with MgO. In addition, it is found that adsorption of CO_2_ on hydrated MgO is slower than that on bare MgO, which means that the presence of H_2_O molecules (moisture environment) can hinder MgO carbonation. In turn, the carbonation of Mg(OH)_2_ is found to be significantly faster than that of MgO. It should be noted that both hydration and carbonation of Mg(OH)_2_ take place at the edges. In addition, a weaker interaction of the CO_2_ and H_2_O molecules with the MgO and Mg(OH)_2_ surfaces compared to the edge and/or defect-containing surfaces (clusters) is found. Importantly, two limiting factors of the HMCs formation reaction are found: (i) surface passivation of Mg(OH)_2_ upon its initial carbonation and (ii) surface covering of Mg(OH)_2_ by H_2_O molecules, which inhibits the carbonation on Mg(OH)_2_.

## Supporting Information Available

Figure S1: various configurations of the CO_2_ molecule on the MgO cluster; Figure S2: adsorption of CO_2_ molecule on the MgO cluster; Figure S3: various configurations of the H_2_O molecule on the MgO cluster; Figure S4: adsorption and dissociation of the H_2_O molecule on MgO cluster; Figure S5: adsorption of the CO_2_ and H_2_O molecules on the MgO cluster; Figure S6: various configurations of the CO_2_ molecule on the hydrated MgO cluster; Figure S7: adsorption of the CO_2_ molecule on the hydrated MgO cluster; Figure S8: various configurations of the CO_2_ molecule on the Mg(OH)_2_ cluster; Figure S9: various configurations of the H_2_O molecule on the Mg(OH)_2_ cluster; Figure S10: various configurations of the H_2_O molecule on the carbonated Mg(OH)_2_ cluster; Figure S11: various configurations of two H_2_O molecules on the carbonated Mg(OH)_2_ cluster; Table S1: adsorption energy *E*_ads_ and the amount of charge transfer Δ*q* between the molecules and the clusters; Table S2: the energy barrier *E*_b_ and the donor/acceptor characteristics of the molecules on the clusters; Table S3: comparison of *E*_ads_ of CO_2_ and H_2_O on bulk and cluster of MgO and Mg(OH)_2_; Table S4: the energy of the isolated molecules and clusters in the gas phase (PDF)
Movie S1: carbonation of the MgO cluster (MP4)
Movie S2: hydration of the MgO cluster (MP4)
Movie S3: carbonation of the hydrated MgO cluster (MP4)
Movie S4: simultaneous carbonation and hydration of the MgO cluster (MP4)
Movie S5: interactions of H_2_O and CO_2_ molecules with the Mg(OH)_2_ cluster (MP4)
Movie S6: interactions of H_2_O and CO_2_ molecules with the carbonated Mg(OH)_2_ cluster (MP4)

## References

[ref1] Kulp S. A.; Strauss B. H. New Elevation Data Triple Estimates of Global Vulnerability to Sea-Level Rise and Coastal Flooding. Nat. Commun. 2019, 10 (1), 1–12. 10.1038/s41467-019-12808-z. 31664024 PMC6820795

[ref2] Olivier J. G. I.; Peters J. A. H. W.; Janssens-Maenhout G. Trends in Global CO_2_ Emissions 2012 Report, 2012.

[ref3] Van Oss H. G.; Padovani A. C. Cement Manufacture and the Environment: Part I: Chemistry and Technology. J. Ind. Ecol. 2002, 6 (1), 89–105. 10.1162/108819802320971650.

[ref4] Birchal V. S. S.; Rocha S. D. F.; Ciminelli V. S. T. The Effect of Magnesite Calcination Conditions on Magnesia Hydration. Miner. Eng. 2000, 13, 1629–1633. 10.1016/S0892-6875(00)00146-1.

[ref5] Walling S. A.; Provis J. L. Magnesia-Based Cements: A Journey of 150 Years, and Cements for the Future?. Chem. Rev. 2016, 116 (7), 4170–4204. 10.1021/acs.chemrev.5b00463. 27002788

[ref6] Gartner E.; Sui T. Alternative Cement Clinkers. Cem. Concr. Res. 2018, 114, 27–39. 10.1016/j.cemconres.2017.02.002.

[ref7] Ruan S.; Unluer C. Comparative Life Cycle Assessment of Reactive MgO and Portland Cement Production. J. Clean. Prod. 2016, 137, 258–273. 10.1016/j.jclepro.2016.07.071.

[ref8] Zhongming Z.; Linong L.; Wangqiang Z.; Wei L. AR6 Climate Change 2021: The Physical Science Basis, 2021.

[ref9] Unluer C.; Al-Tabbaa A. Impact of Hydrated Magnesium Carbonate Additives on the Carbonation of Reactive MgO Cements. Cem. Concr. Res. 2013, 54, 87–97. 10.1016/j.cemconres.2013.08.009.

[ref10] Li X. Mechanical Properties and Durability Performance of Reactive Magnesia Cement Concrete; University of Cambridge: 2013.

[ref11] Ma S.; Akca A. H.; Esposito D.; Kawashima S. Influence of Aqueous Carbonate Species on Hydration and Carbonation of Reactive MgO Cement. J. CO_2_ Util. 2020, 41, 101260 10.1016/j.jcou.2020.101260.

[ref12] Unluer C.; Al-Tabbaa A. Enhancing the Carbonation of MgO Cement Porous Blocks through Improved Curing Conditions. Cem. Concr. Res. 2014, 59, 55–65. 10.1016/j.cemconres.2014.02.005.

[ref13] Thomas J. J.; Musso S.; Prestini I. Kinetics and Activation Energy of Magnesium Oxide Hydration. J. Am. Ceram. Soc. 2014, 97 (1), 275–282. 10.1111/jace.12661.

[ref14] Matabola K. P.; van der Merwe E. M.; Strydom C. A.; Labuschagne F. J. W. The Influence of Hydrating Agents on the Hydration of Industrial Magnesium Oxide. J. Chem. Technol. Biotechnol. 2010, 85 (12), 1569–1574. 10.1002/jctb.2467.

[ref15] Dung N. T.; Unluer C. Development of MgO Concrete with Enhanced Hydration and Carbonation Mechanisms. Cem. Concr. Res. 2018, 103, 160–169. 10.1016/j.cemconres.2017.10.011.

[ref16] Zhao J.; Huang X.; Shi R.; Tang L.; Su Y.; Sai L. Ab Initio Global Optimization of Clusters. Chem. Model. 2015, 12, 249–292. 10.1039/9781782622703-00249.

[ref17] Kistanov A. A.; Shcherbinin S. A.; Ustiuzhanina S. V.; Huttula M.; Cao W.; Nikitenko V. R.; Prezhdo O. V. First-Principles Prediction of Two-Dimensional B3C2P3 and B2C4P2: Structural Stability, Fundamental Properties, and Renewable Energy Applications. J. Phys. Chem. Lett. 2021, 12 (13), 3436–3442. 10.1021/acs.jpclett.1c00411. 33789049

[ref18] Liu B.; Zhou K. Recent Progress on Graphene-Analogous 2D Nanomaterials: Properties, Modeling and Applications. Prog. Mater. Sci. 2019, 100, 99–169. 10.1016/j.pmatsci.2018.09.004.

[ref19] Alessio M.; Usvyat D.; Sauer J. Chemically Accurate Adsorption Energies: CO and H_2_O on the MgO (001) Surface. J. Chem. Theory Comput. 2019, 15 (2), 1329–1344. 10.1021/acs.jctc.8b01122. 30596490

[ref20] Fan H.-X.; Cui T.-Y.; Rajendran A.; Yang Q.; Feng J.; Yue X.-P.; Li W.-Y. Comparative Study on the Activities of Different MgO Surfaces in CO_2_ Activation and Hydrogenation. Catal. Today 2020, 356, 535–543. 10.1016/j.cattod.2020.03.010.

[ref21] Huang J.; Li X.; Wang X.; Fang X.; Wang H.; Xu X. New Insights into CO_2_Methanation Mechanisms on Ni/MgO Catalysts by DFT Calculations: Elucidating Ni and MgO Roles and Support Effects. J. CO_2_ Util. 2019, 33, 55–63. 10.1016/j.jcou.2019.04.022.

[ref22] Jang J. M.; Kang S. G. Understanding CO_2_ Adsorption on a M1 (M2)-Promoted (Doped) MgO–CaO (100) Surface (M1= Li, Na, K, and Rb, M2= Sr): A DFT Theoretical Study. ACS Sustain. Chem. Eng. 2019, 7 (20), 16979–16984. 10.1021/acssuschemeng.9b01223.

[ref23] Cornu D.; Guesmi H.; Krafft J.-M.; Lauron-Pernot H. Lewis Acido-Basic Interactions between CO_2_ and MgO Surface: DFT and DRIFT Approaches. J. Phys. Chem. C 2012, 116 (11), 6645–6654. 10.1021/jp211171t.

[ref24] Wu S.; Tan B. T.; Senevirathna H. L.; Wu P. Polarization of CO_2_ for Improved CO_2_ Adsorption by MgO and Mg (OH)_2_. Appl. Surf. Sci. 2021, 562, 150187 10.1016/j.apsusc.2021.150187.

[ref25] Dung N. T.; Lesimple A.; Hay R.; Celik K.; Unluer C. Formation of Carbonate Phases and Their Effect on the Performance of Reactive MgO Cement Formulations. Cem. Concr. Res. 2019, 125, 105894 10.1016/j.cemconres.2019.105894.

[ref26] Hopkinson L.; Kristova P.; Rutt K.; Cressey G. Phase Transitions in the System MgO–CO_2_–H_2_O during CO_2_ Degassing of Mg-Bearing Solutions. Geochim. Cosmochim. Acta 2012, 76, 1–13. 10.1016/j.gca.2011.10.023.

[ref27] Castleman A. W.; Bowen Kh Clusters: Structure, Energetics, and Dynamics of Intermediate States of Matter. J. Phys. Chem. 1996, 100 (31), 12911–12944. 10.1021/jp961030k.

[ref28] Ohlin C. A.; Villa E. M.; Rustad J. R.; Casey W. H. Dissolution of Insulating Oxide Materials at the Molecular Scale. Nat. Mater. 2010, 9 (1), 11–19. 10.1038/nmat2585. 20019664

[ref29] Kresse G.; Furthmüller Efficient Iterative Schemes For Ab Initio Total-Energy Calculations Using a Plane-Wave Basis Set. J. Phys. Rev. B: Condens. Matter Mater. Phys. 1996, 54, 11169–11186. 10.1103/PhysRevB.54.11169. 9984901

[ref30] Blöchl P. E. Projector Augmented-Wave Method. Phys. Rev. B 1994, 50 (24), 17953 10.1103/PhysRevB.50.17953. 9976227

[ref31] Perdew J. P.; Burke K.; Ernzerhof M. Generalized Gradient Approximation Made Simple. Phys. Rev. Lett. 1996, 77 (18), 3865 10.1103/PhysRevLett.77.3865. 10062328

[ref32] Zhang S.; Zhang Y.; Huang S.; Liu H.; Wang P.; Tian H. Theoretical Investigation of Growth, Stability, and Electronic Properties of Beaded ZnO Nanoclusters. J. Mater. Chem. 2011, 21 (42), 16905–16910. 10.1039/c1jm12061a.

[ref33] Chen M.; Dixon D. A. Structure and Stability of Hydrolysis Reaction Products of MgO Nanoparticles Leading to the Formation of Brucite. J. Phys. Chem. C 2017, 121 (39), 21750–21762. 10.1021/acs.jpcc.7b07507.

[ref34] Zhang D.; Sun Y.; Chen L.; Zhang S.; Pan N. Influence of Fabric Structure and Thickness on the Ballistic Impact Behavior of Ultrahigh Molecular Weight Polyethylene Composite Laminate. Mater. Des. 2014, 54, 315–322. 10.1016/j.matdes.2013.08.074.

[ref35] Becke A. D. Density-Functional Exchange-Energy Approximation with Correct Asymptotic Behavior. Phys. Rev. A 1988, 38 (6), 3098 10.1103/PhysRevA.38.3098. 9900728

[ref36] Niu J.; Wang Y.; Qi Y.; Dam A. H.; Wang H.; Zhu Y.-A.; Holmen A.; Ran J.; Chen D. New Mechanism Insights into Methane Steam Reforming on Pt/Ni from DFT and Experimental Kinetic Study. Fuel 2020, 266, 117143 10.1016/j.fuel.2020.117143.

[ref37] Richard F. W. Bader Atoms in Molecules – A Quantum Theory; Oxford University Press: New York, 1990.

[ref38] Laidler K. J. Chemical Kinetics; Harper & Row: New York, 1987.

[ref39] Kistanov A. A.; Cai Y.; Zhou K.; Srikanth N.; Dmitriev S. V.; Zhang Y.-W. Exploring the Charge Localization and Band Gap Opening of Borophene: A First-Principles Study. Nanoscale 2018, 10 (3), 1403–1410. 10.1039/C7NR06537J. 29302656

[ref40] Henkelman G.; Uberuaga B. P.; Jónsson H. A Climbing Image Nudged Elastic Band Method for Finding Saddle Points and Minimum Energy Paths. J. Chem. Phys. 2000, 113 (22), 9901–9904. 10.1063/1.1329672.

[ref41] Nosé S. A Unified Formulation of the Constant Temperature Molecular Dynamics Methods. J. Chem. Phys. 1984, 81 (1), 511–519. 10.1063/1.447334.

[ref42] Jensen M. B.; Pettersson L. G. M.; Swang O.; Olsbye U. CO_2_ Sorption on MgO and CaO Surfaces: A Comparative Quantum Chemical Cluster Study. J. Phys. Chem. B 2005, 109 (35), 16774–16781. 10.1021/jp052037h. 16853136

[ref43] Dung N. T.; Unluer C. Performance of Reactive MgO Concrete under Increased CO_2_ Dissolution. Cem. Concr. Res. 2019, 118, 92–101. 10.1016/j.cemconres.2019.02.007.

[ref44] Unluer C. Carbon Dioxide Sequestration in Magnesium-Based Binders; Woodhead Publishing. 2018; pp 129–173.

[ref45] Mutisya S. M.; Kalinichev A. G. Carbonation Reaction Mechanisms of Portlandite Predicted from Enhanced Ab Initio Molecular Dynamics Simulations. Minerals 2021, 11 (5), 509 10.3390/min11050509.

