# Supplementary material for: Exploring Mechanisms of Hydration and Carbonation of MgO and Mg(OH)2 in Reactive Magnesium Oxide-Based Cements
Source: J Phys Chem C Nanomater Interfaces. 2022 Mar 30;126(14):6196–206. doi: 10.1021/acs.jpcc.1c10590 (PMC9014411; doi:10.1021/acs.jpcc.1c10590)
Supplement: Supplementary file 1 — jp1c10590_si_001.pdf [file jp1c10590_si_001.pdf]

Supporting Information

Exploring Mechanisms of Hydration and Carbonation of MgO and $Mg(OH)_2$ in Reactive Magnesium Oxide-based Cements

Mina Ghane Gardeh,[a] Andrey A. Kistanov,[*,b] Hoang Nguyen,[a] Hegoi Manzano,[c] Wei Cao,[b] and Paivo Kinnunen[a]

[a]*Fibre and Particle Engineering Research Unit, University of Oulu, Pentti Kaiteran katu 1, 90014 Oulu, Finland.*

[b]*Nano and Molecular Systems Research Unit, University of Oulu, Pentti Kaiteran katu 1, 90014 Oulu, Finland.*

[c]*Departament of Condensed Matter Physics, University of the Basque Country (UPV/EHU), Barrio Sarriena, s/n, 48940 Leioa, Spain.*

**Corresponding author: andrey.kistanov@oulu.fi*

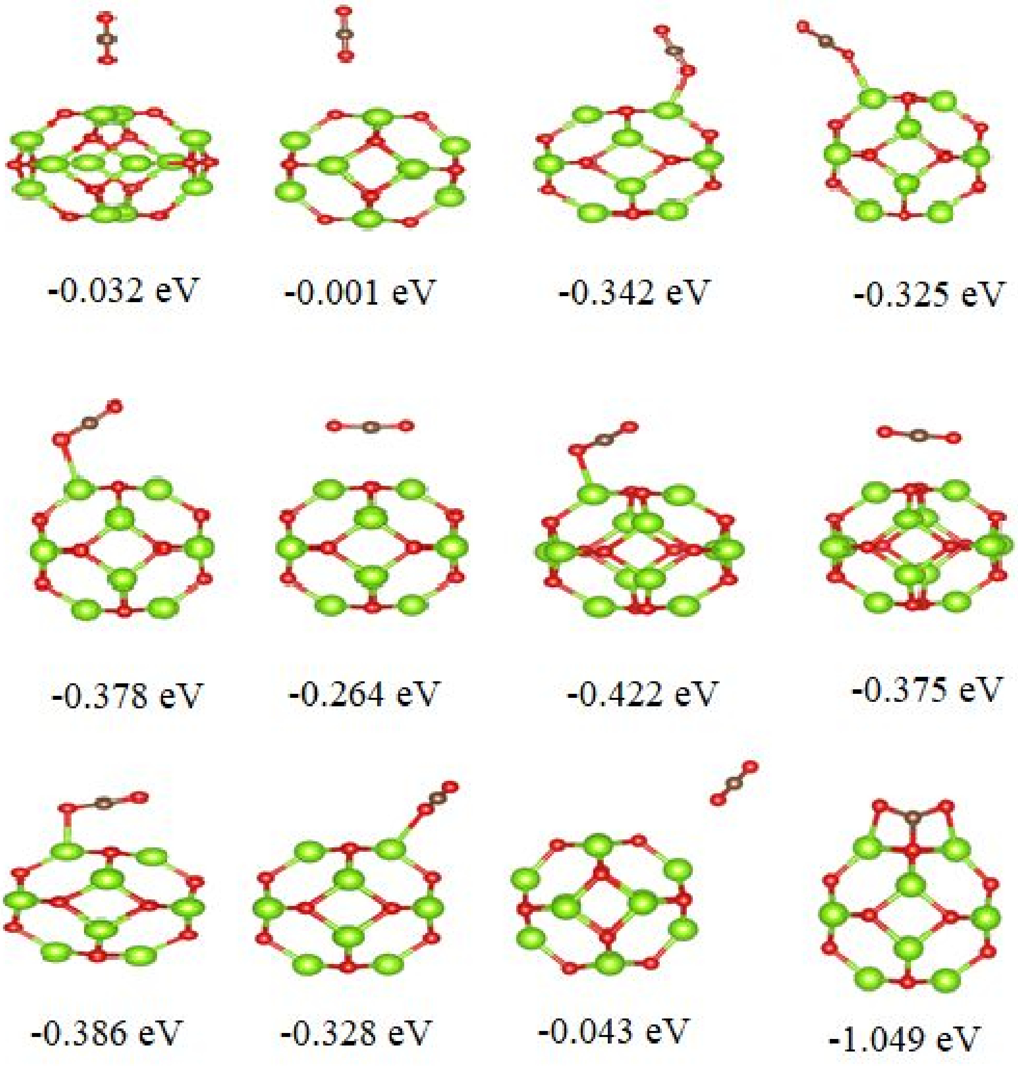

Figure S1. Various configurations of the CO_2 molecule on the MgO cluster.

Carbonation of MgO cluster. According to atomic trajectories for one set of the AIMD simulation (Figure S1a and b), the time needed for the physisorption of CO_2 on MgO is 2.4 ps, while the chemisorption occurs in 2.7 ps.

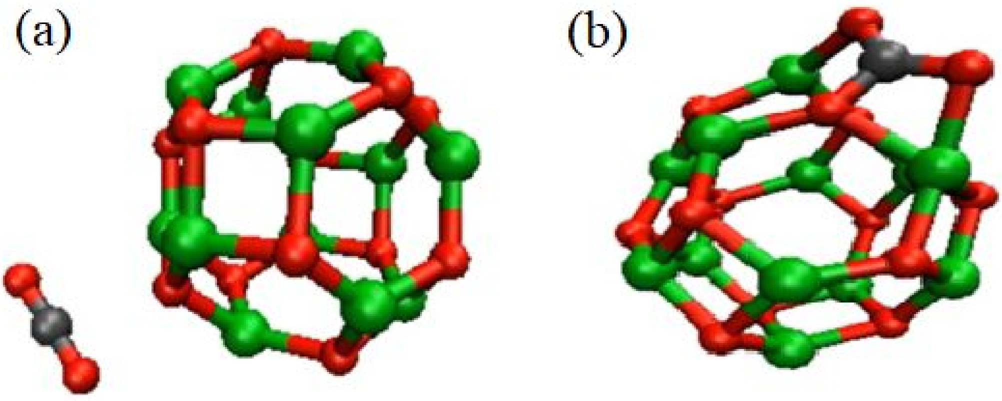

Figure S2. (a) Physisorbed CO_2 molecule on MgO cluster (2.4 ps). (b) Chemisorbed CO_2 molecule on MgO cluster (2.7 ps).

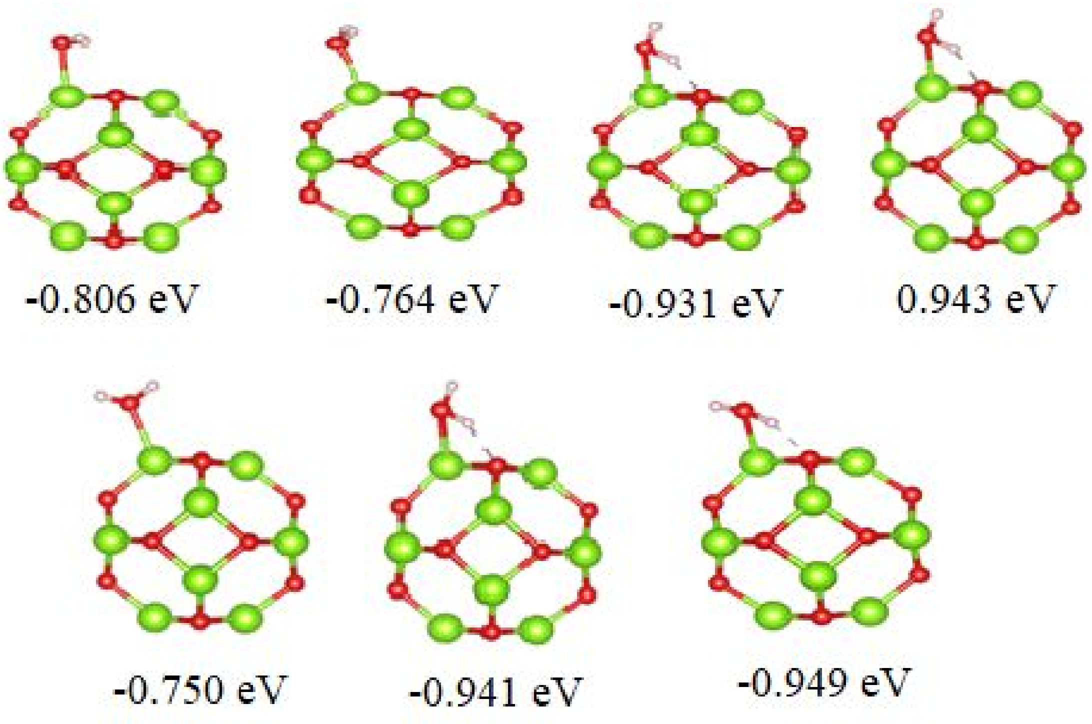

Figure S3. Various configurations of the H_2O molecule on the MgO cluster.

Hydration of MgO cluster. AIMD simulations confirm that H_2O molecule is dissociated on the MgO cluster in 0.9 ps (Figure S2). Therefore, the required time for the hydration of the MgO cluster (0.9 ps) is 3 times lower than the time needed for its carbonation (2.7 ps).

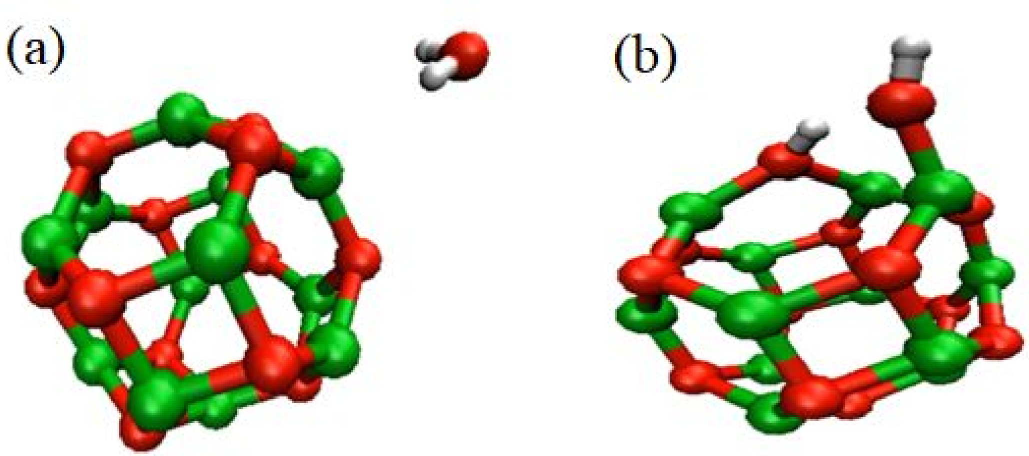

Figure S4. (a) Physisorbed H_2O molecule on MgO cluster. (b) dissociated H_2O molecule on MgO cluster (0.9).

Comparison of carbonation and hydration of MgO cluster. To compare the carbonation and hydration rates of MgO, three CO_2 molecules and three H_2O molecules are placed on the MgO cluster, as it is seen in Movie 3, the first, the second, and the third H_2O molecules are bonding to the cluster in 0.2 ps, 0.7 ps, and 1.5 ps, respectively. Whereas the physisorption and chemisorption of the first CO_2 molecule occurs in 2.2 ps, and 2.5 ps, respectively. Two other CO_2 molecules does not adsorb on the MgO cluster in the considered period of time. As a result, carbonation of MgO cluster occurs much slower than its hydration.

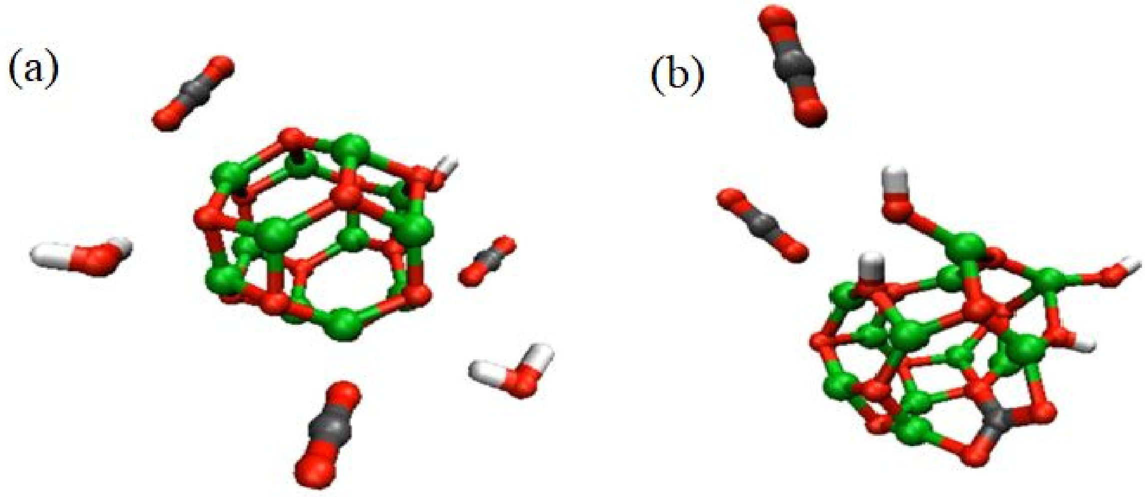

Figure S5. (a) Physisorbed CO_2 and H_2O molecules on the MgO cluster. (b) Chemisorbed CO_2 and H_2O molecules on the MgO cluster.

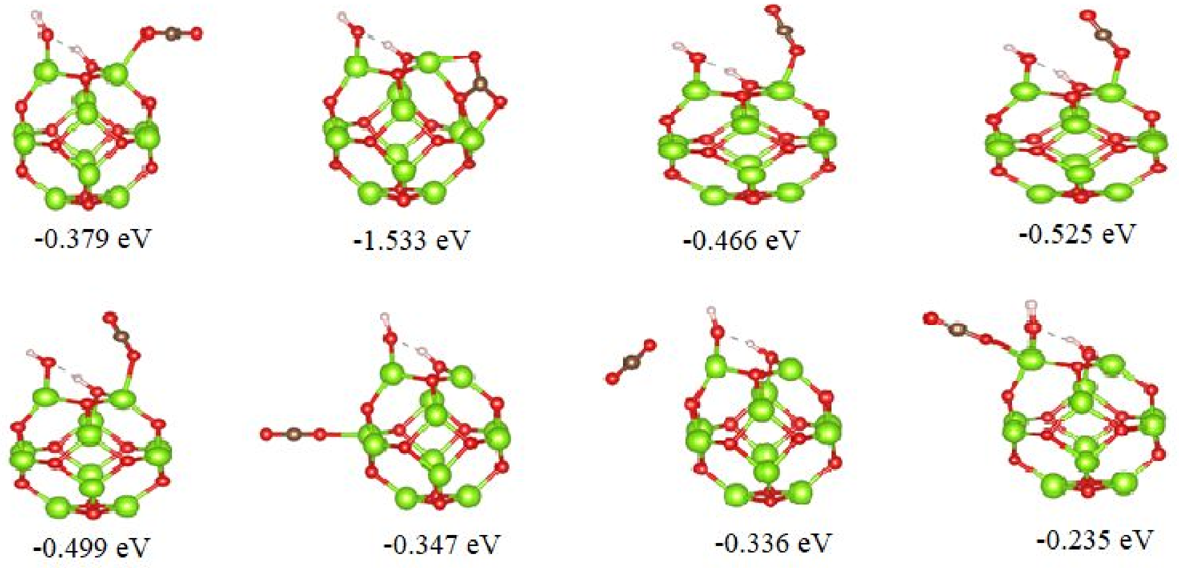

Figure S6. Various configurations of the CO_2 molecule on the hydrated MgO cluster.

Carbonation of hydrated MgO cluster. AIMD simulations (Figure S4) show that the time of physisorption and chemisorption of the CO_2 molecule on hydrated MgO cluster is 4.2 ps and 4.3 ps, respectively. Comparing to carbonation of bare MgO (2.6 ps), carbonation of hydrated MgO (4.3 ps) takes longer time (Figure S1).

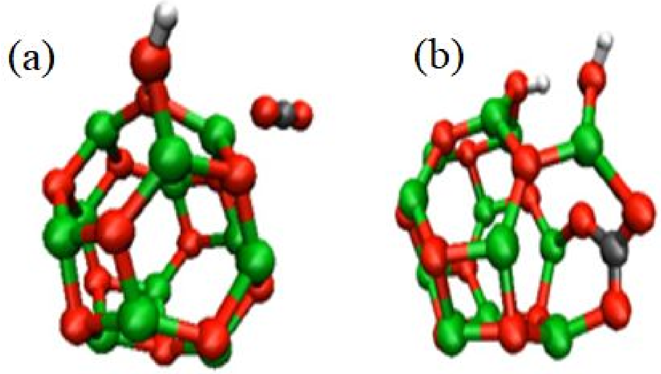

Figure S7. (a) Physisorbed CO_2 molecule on the hydrated MgO cluster (2.6 ps). (b) Chemisorbed CO_2 molecule on the hydrated MgO cluster (4.3 ps).

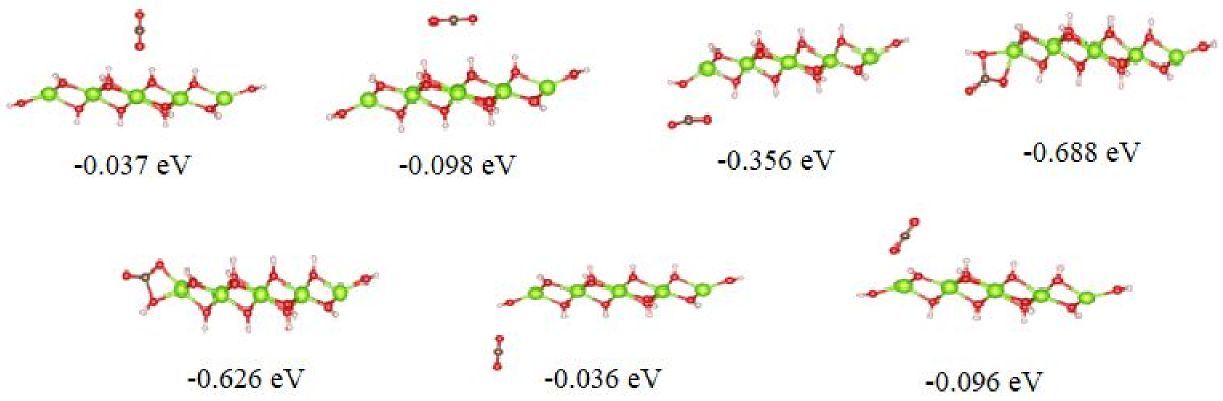

Figure S8. Various configurations of the CO_2 molecule on the $Mg(OH)_2$ cluster.

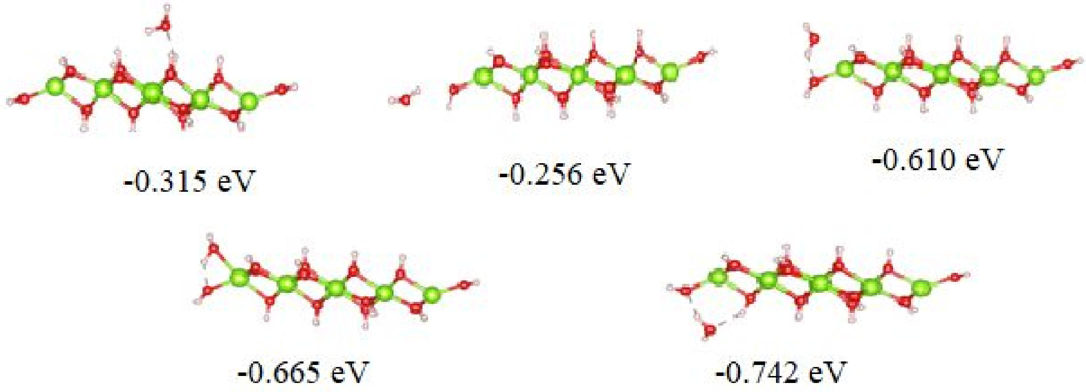

Figure S9. Various configurations of the H_2O molecule on the $Mg(OH)_2$ cluster.

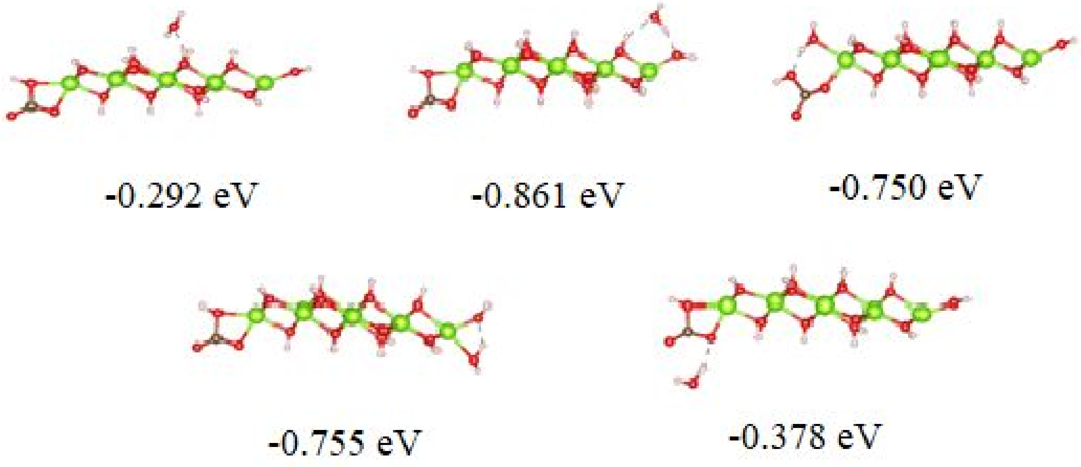

Figure S10. Various configurations of the H_2O molecule on the carbonated $Mg(OH)_2$ cluster.

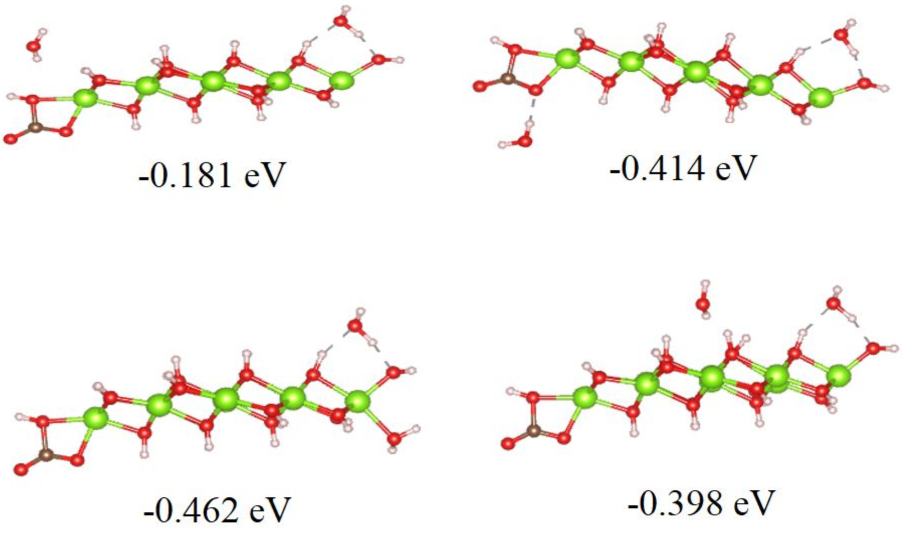

Figure S11. Various configurations of two H_2O molecules on the carbonated $Mg(OH)_2$ cluster.

Table S1. Adsorption energy E_{ads} and the amount of charge transfer Δq between the molecules and the clusters. A negative/positive Δq indicates a gain /loss of electrons from each molecule to the cluster.

**Molecule**	**MgO**		**$Mg(OH)_2$**	
	**E_{ads}, eV**	**Δq, e per molecule**	**E_{ads}, eV)**	**Δq, e per molecule**
**CO_2(Physisorbed)**	-0.42	-0.092	-	-
**CO_2(Chemisorbed)**	-1.05	-0.117	-0.69	-0.397
**H_2O**	-0.95	-1.122	-0.74	-0.044
**$CO_2 + H_2O$ (Physisorbed)**	-0.53	-0.037	-	-
**$CO_2 +$ H_2O(Chemisorbed)**	-1.55	-0.086	-0.86	-0.046
**$CO_2 + 2H_2O$**	-	-	-0.46	-0.037

Table S2. The energy barrier E_b and the donor/acceptor characteristics of the molecules on the clusters.

**Molecule**	**MgO**		**$Mg(OH)_2$**	
	**E_b, eV**	**Acceptor/Donor**	**E_b, eV**	**Acceptor/Donor**
**CO_2(physisorbed)**	-	Acceptor	-	Acceptor
**CO_2(Chemisorbed)**	0.049	Acceptor	0.002	Acceptor
**H_2O**	0.245	Acceptor	-	Acceptor
**$CO_2 + H_2O$**	0.275	Acceptor (CO_2)	-	Acceptor (H_2O)
**$CO_2 + 2H_2O$**	-	-	-	Acceptor (H_2O)

Table S3. The comparison of E_{ads} of CO_2 and H_2O on bulk and cluster of MgO and $Mg(OH)_2$.

**Molecule**	**MgO**		**$Mg(OH)_2$**	
	**E_{ads}, eV (cluster)**	**E_{ads}, eV (bulk)**	**E_{ads}, eV (cluster)**	**E_{ads}, eV (bulk)**
**CO_2(Physisorbed)**	-0.42	-0.34	-	-
**CO_2(Chemisorbed)**	-1.05	-	-0.69	-0.25
**H_2O**	-0.95	-0.58	-0.74	-0.37

Table S4. The energy of the isolated molecules and clusters in the gas phase.

**Structure**	**CO_2**	**H_2O**	**MgO**	**$Mg(OH)_2$**
**Energy, eV**	-21.56	-13.42	-118.45	-224.08
